# Cadmium Uptake, *MT* Gene Activation and Structure of Large-Sized Multi-Domain Metallothioneins in the Terrestrial Door Snail *Alinda biplicata* (Gastropoda, Clausiliidae)

**DOI:** 10.3390/ijms21051631

**Published:** 2020-02-27

**Authors:** Veronika Pedrini-Martha, Simon Köll, Martin Dvorak, Reinhard Dallinger

**Affiliations:** Department of Zoology and Center of Molecular Biosciences Innsbruck, University of Innsbruck, Technikerstraße 25, 6020 Innsbruck, Austria; Simon.Koell@student.uibk.ac.at (S.K.); martin.dvorak@uibk.ac.at (M.D.)

**Keywords:** multi-domain metallothionein, cadmium metabolism, RNA expression, intronless genes, *Alinda biplicata*, Gastropoda, avoidance behavior

## Abstract

Terrestrial snails (Gastropoda) possess Cd-selective metallothioneins (CdMTs) that inactivate Cd^2+^ with high affinity. Most of these MTs are small Cysteine-rich proteins that bind 6 Cd^2+^ equivalents within two distinct metal-binding domains, with a binding stoichiometry of 3 Cd^2+^ ions per domain. Recently, unusually large, so-called multi-domain MTs (md-MTs) were discovered in the terrestrial door snail *Alinda biplicata (A.b.)*. The aim of this study is to evaluate the ability of *A.b.* to cope with Cd stress and the potential involvement of md-MTs in its detoxification. Snails were exposed to increasing Cd concentrations, and Cd-tissue concentrations were quantified. The gene structure of two *md-MTs* (*9md-MT* and *10md-MT*) was characterized, and the impact of Cd exposure on *MT* gene transcription was quantified via qRT PCR. *A.b.* efficiently accumulates Cd at moderately elevated concentrations in the feed, but avoids food uptake at excessively high Cd levels. The structure and expression of the long *md-MT* genes of *A.b.* were characterized. Although both genes are intronless, they are still transcribed, being significantly upregulated upon Cd exposure. Overall, our results contribute new knowledge regarding the metal handling of *Alinda biplicata* in particular, and the potential role of md-MTs in Cd detoxification of terrestrial snails, in general.

## 1. Introduction

Gastropods represent the most species-rich class within the phylum of Mollusca. Throughout evolution, some gastropod lineages left their marine realms and colonized limnic and terrestrial habitats. The common door snail, *Alinda biplicata* (family of Clausiliidae), belongs to the gastropod clade of Stylommatophora that has successfully adapted to a terrestrial lifestyle. *A. biplicata* is widely distributed in central Europe and can be found in forests, between rocks in urban areas (e.g., parks) or at riverside banks [1]. It is one of the rare ovoviviparous snail species in which the whole embryonic development takes place inside the egg within the reproductive tract of the adult individual [2]. The completion of the shell growth of hatched juvenile snails needs 20 to 28 weeks. Subsequently, it takes another 24 to 36 weeks until readily grown snails finish maturation and start to reproduce [2]. To ensure its survival under harsh and sometimes rapidly changing environmental conditions, *A. biplicata* relies on innate protective stress response mechanisms. For example, a proven strategy to overcome potentially stressful periods is its ability to switch to a state of inactivity, the so-called aestivation [3,4]. Another protective feature of terrestrial snails is their ability to highly accumulate and inactivate toxic metal ions such as Cadmium (Cd) in their digestive organs and cells [5,6].

One of the most important physiological protection mechanisms involved in metal accumulation and detoxification of snails is the expression of metal-binding proteins of the metallothionein (MT) family [6,7,8]. MTs are cysteine-rich, metal-binding proteins primarily responsible for cellular metal homeostasis and detoxification [9,10,11]. MTs may also be involved in protection against oxidative stress [12] and, more generally, in stress response reactions [13,14,15,16]. Mono- and divalent metal ions such as Zn^2+^, Cd^2+^, or Cu^+^ are bound by MTs through the sulfhydryl groups of cysteines arranged in highly conserved Cys-Xaa-Cys motifs, building firm metal thiolate clusters [17]. As a particular feature, many snail species express metal-selective MT variants and isoforms derived from metal-unspecific precursors in basal gastropod ancestors [18,19]. Metal-selective snail MTs were first discovered in the helicid snails *Helix pomatia* and *Cornu aspersum*. Typically, these MTs not only exhibit a metal-selective binding behavior but are also characterized by their distinct cellular expression patterns and physiological functions directly related to the selectively bound metal ion [6,20,21,22]. For example, Cu-selective MT isoforms (CuMT) of snails preferentially bind monovalent Cu ions, being expressed in a particular cell type called rhogocyte, involved in Cu homeostasis and hemocyanin turnover [21,23]. A second, Cd-selective MT isoform (CdMT) binds Cd^2+^ with high affinity and is devoted to Cd-detoxification and stress response [7,20]. In addition, a third, non-metal-selective MT isoform (Cd/CuMT) is able to bind both metal ions simultaneously [6,24], being involved in embryonic snail development [22]. Most snail MTs are structurally organized in two metal-binding domains (N- and C-terminal) connected through a short linker region, exhibiting a binding stoichiometry of six divalent (Cd^2+^, Zn^2+^) or twelve monovalent (Cu^+^) metal ions [23,25]. More recent studies demonstrate, however, a greater variability of gastropod MT protein structures than so far known. The marine periwinkle *Littorina littorea* and its terrestrial relative *Pomatias elegans*, for example, have increased the metal-binding capacity of their Cd-selective MTs by addition of a third Cd-binding domain, derived from a duplication of their N-terminal domain [26,27]. Consequently, these newly discovered three-domain MTs have increased their binding stoichiometry from 6 to 9 Cd^2+^ ions [26,28]. Our most recent studies indicate the presence of multi-domain MTs (md-MTs) with as much as eight (8) metal-binding domains (8md-MT) in an MT of the freshwater snail *Marisa cornuarietis*, and with nine (9) or even ten (10) metal-binding domains (9md-MT and 10md-MT) in two MT isoforms of the terrestrial snail *A. biplicata* [19]. Following the findings of increased Cd-binding capacities in the three-domain MTs of *Littorina littorea* and *Pomatias elegans* (see above), we suggest that the additional multiplication of N-terminal metal-binding domains in the MTs of *Marisa cornuarietis* and *A. biplicata* might increase their metal ion stoichiometry and hence, their metal-binding capacity to a correspondingly high level. These new findings remind of molecular evolutionary mechanisms in other species such as *Drosophila melanogaster*, where an increased Cd resistance in some populations has been achieved by *MT* gene duplication events [29].

In the present study, we will explore how the terrestrial snail *A. biplicata* can cope with Cd stress and which mechanisms might be involved in this process. In particular, we will focus on the transcriptional activation of *9md-MT*, *10 md-MT*, and of an additional two-domain *CuMT* gene of this snail and their potential role in Cd accumulation and detoxification. Therefore, adult individuals of *A. biplicata* were exposed to Cd in the laboratory. First, the *MT* gene structure of the large *9md-MT* and *10md-MT* isoforms was characterized and analyzed for the first time. In addition, multiple parameters such as the feeding behavior of metal-exposed snails, their Cd tissue accumulation, and the transcriptional upregulation of the two multi-domain *MT* genes (*9md-MT* and *10md-MT*) and the *CuMT* gene were quantified and evaluated.

## 2. Results and Discussion

### 2.1. Cd Accumulation in Alinda biplicata Is Restrained beyond a High Threshold Level

**Figure 1:**
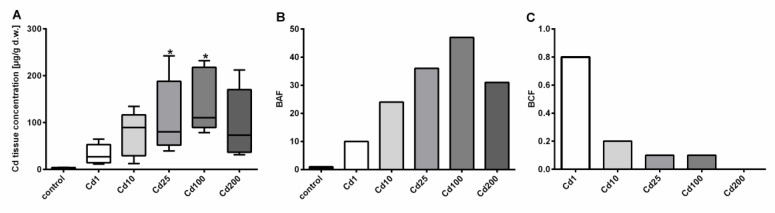
Dose-dependent Cd accumulation in *A. biplicata* upon Cd exposure through 4 days. (**A**) Whisker box plots showing Cd soft-tissue concentrations in µg/g d.w. of controls (*n* = 4) and Cd-treated snails (*n* = 5 each) from the lowest to the highest value of each treatment group. The respective box extends from the 25th to the 75th percentile, with the square line showing the median. Asterisks (*) indicate significance (*p* < 0.05) compared to control value. (**B**) Bar graph showing the bioaccumulation factor (BAF) of respective treatment groups, reflecting the metal accumulation in soft tissues of treated snails, referred to controls (BAF = 1). (**C**) Bar graph representing the bioconcentration factor (BCF; for calculation see Material and Methods) for all Cd exposure groups. The measured Cd concentrations of snail cookies of each treatment group were as follows: Cd1 = 38; Cd10 = 355.3; Cd25 = 804.5; Cd100 = 2692.6; Cd200 = 6396.1 µg/g d.w.

After a short-term accumulation period of 4 days, a dose-dependent Cd accumulation was observed in the soft-tissues of *A. biplicata* up to an exposure concentration of 2692.6 µg/g d.w. Cd in the food (Cd100), but the accumulation did not further increase beyond this threshold (Figure 1).

Cd concentrations in soft tissue of control snails ranged between 2.14 and 4.49 µg/g d.w., whereas the highest Cd load was assessed in snails exposed to 2693 µg/g d.w. Cd (Cd100), where the median Cd tissue concentration reached 145.12 µg/g d.w. (Figure 1). At the highest Cd exposure level of 6396 µg/g d.w. (Cd200), no further increase in Cd tissue accumulation was observed (Figure 1), suggesting that the accumulation curve might have reached a plateau level. Interestingly, no difference in mortality between most treatment groups (*n* = 10 each) was detected (1 to 2 dead snails per treatment group) except for the highest treatment group (Cd200), where three snails out of 10 died during the experiment. It is questionable, however, whether this indicates a significant increase in mortality. In addition, many snails in the highest treatment groups switched to a state of aestivation, which made it more difficult to distinguish between dead and alive individuals. Overall, it is probable that Cd exposure regimes applied did not reach lethal concentration levels, which suggests that *A. biplicata* must be highly tolerant against Cd exposure. Even though not statistically significant, an increasing trend of Cd accumulation of the two low Cd-exposure groups is apparent. A possible reason for the lacking significance in these cases may be the high variability of Cd soft-tissue concentrations within the respective treatment groups (Figure 1A), most likely owing to differing degrees of activity and feeding rates of individual snails [14]. Whereas Cd feed concentrations of 38 and 355 µg/g Cd d.w. in the two lowest treatment groups (Cd1 and Cd10) can be compared with concentrations of highly metal-polluted terrestrial habitats, this is certainly not the case for the higher Cd treatments (Cd25, Cd100, and Cd200) in the present study. Cd soil concentrations of most terrestrial habitats do not exceed the range of 1 to 2 µg/g Cd within Europe [30], but at highly contaminated sites, Cd soil content can increase up to 232 µg/g d.w. [31]. Cd concentrations of litter can also vary to a great extent, and highly elevated Cd litter concentrations at contaminated sites can be detected [32,33].

Evidently, *A. biplicata* possesses a marked capacity for Cd bioaccumulation, as shown in the present study by the high Cd concentrations reached in the soft tissue of metal-exposed snails, in which Cd can be concentrated up to 47-fold referred to control individuals (Figure 1B). However, when analyzing the bioconcentration factor (BCF), *A. biplicata* seems not to be a “macroconcentrator” species for Cd (Figure 1C) in contrast to its near relative species like *Helix pomatia*, *Arianta arbustorum,* or *Cornu aspersum,* being able to accumulate Cd from the environment, with BCF values above 2 [34]. However, this observation is preliminary, considering that within the short exposure period of 4 days in the present study, steady state levels for Cd bioconcentration might not yet have been reached [34]. Notwithstanding the above, it appears that most terrestrial snail species (including probably *A. biplicata*) are suitable organisms for monitoring Cd contamination of their respective soil habitats [5,13,35].

### 2.2. Avoidance Behavior

**Figure 2:**
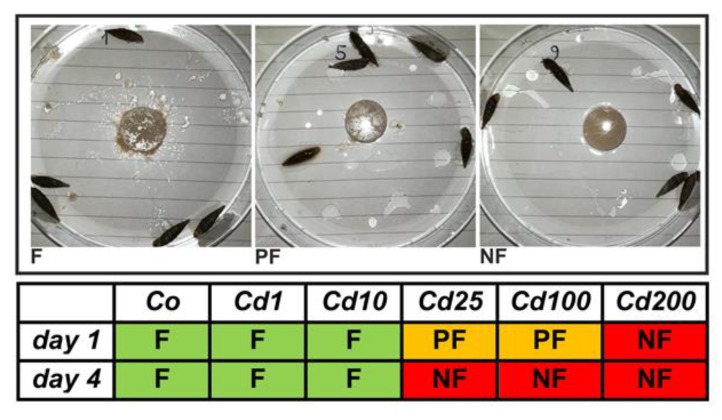
Feeding behavior of *A. biplicata* in control snails and individuals fed on Cd-contaminated cookies. The photographs illustrate the three categories of feeding (F), poor feeding (PF), and non-feeding (NF) in control (Co) and Cd-exposed snails (Cd1, Cd10, Cd25, Cd100, and Cd200) (for exact definition of feeding groups, see text). Cd exposure groups were defined as shown in the legend of Figure 1.

Snails of all treatment groups were photographed before and after feeding. Three different feeding categories could be distinguished (Figure 2): snails which fed regularly (F) by showing clear feeding marks on the respective snail cookies, and with parts of the cookies being scattered through multiple sites within the petri dishes. When clearly visible feeding marks on snail cookies were lacking but small parts of them were distributed within the petri dishes, snails were categorized as poor feeders (PF). Treatment groups were defined as non-feeding (NF) snails when both clear visible feeding marks and randomly distributed cookie parts within petri dishes were absent.

The observed feeding activity of snails decreased at higher Cd exposure levels (Figure 2). Controls and individuals of lower Cd treatment groups (Cd1 with 38 and Cd10 with 355 µg/g Cd) showed an active feeding behavior (Figure 2). Snails treated with higher Cd concentrations (Cd25 with 804.5 and Cd100 with 2692.6 µg/g Cd) started to feed poorly at the beginning of the experiment and stopped feeding during the end of the experiment. No feeding activity at all was observed for snails of the highest treatment group (Cd200 with 6396.1 µg/g Cd), even though crawling tracks were present on the surface of the respective snail cookies (Figure S1). However, individuals of this treatment group accumulated Cd, too (Figure 1). The reason for this must evidently be Cd absorption through the skin of the snail’s foot. In fact, dermal uptake of Cd and other toxic metals has frequently been reported in land snails [36,37,38]. Our data indicate that at lower Cd exposure concentrations, the detoxification system of *A. biplicata* may be sufficient for handling the apparent dietary Cd load. Hence, no behavioral adaptation like a reduction of feeding activity is necessary. However, when Cd exposure exceeds a certain threshold concentration, the capacity of the detoxification system is apparently overstrained and, consequently, snails adapt an avoidance behavior and/or switch into a state of inactivity, the so-called aestivation, to save themselves from severe permanent damage caused by excessive Cd uptake. The reduction of food consumption to avoid metal uptake has been documented for other gastropod species, too, including terrestrial snails like *Cornu aspersum* [39], *Cepaea nemoralis* [40], *Theba pisana* [41], and *Oxyloma pfeifferi* [42], or freshwater species such as *Physella columbiana* [43] and *Lymnaea stagnalis* [44]. Occasionally, this adaptive behavioral response may be inherited by snails that experienced metal pollution during their life cycle to their offspring [43]. In some cases, metal-exposed snails not only show a reduced feeding activity but even start to aestivate [14]. Changing to aestivation could have several benefits such as avoiding or limiting the uptake of toxic metal ions, but also restoring cellular health by fasting. It was demonstrated that in the periwinkle *Littorina littorea*, fasting can increase lysosomal stability, decrease lipid peroxidation (LPO), and induce autophagic cytoprotective mechanisms to remove degraded or harmful protein aggregates [45].

### 2.3. Characterization of Multi-Domain MT Genes in Alinda Biplicata

**Figure 3:**
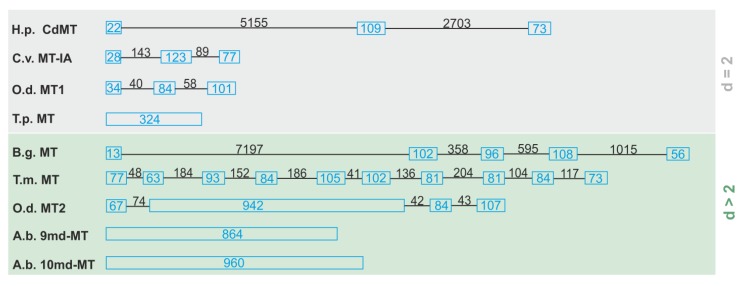
Outline of different gene structures of two (d = 2) and multi-domain (d > 2) MTs. Blue boxes represent the exons, introns are symbolized by lines. The exact length of exons and introns is given in bp (blue for exons, black for introns). Species abbreviations and references (in brackets) are as follows: O.d., *Oikopleura dioica* [47]; H.p., *Helix pomatia* [13]; B.g., *Biomphalaria glabrata* [48]; A.b., *Alinda biplicata* (this study); T.m., *Tremella mesenterica* [49]; C.v., *Crassostrea virginica* [50]; T.p., *Tetrahymena pyriformis* [51].

The gene structure of the two novel *md-MTs* (*9md-MT* and *10md-MT*) of this species was disclosed for the first time in the present study and characterized over a stretch from the start to the stop codon. The most surprising feature of the *9md-MT* and *10md-MT* genes of *A. biplicata* is their lack of introns (Figure 3), resulting in a gene length of 864 bp for the *9md-MT* gene (GenBank MT084760) and 960 bp for the *10md-MT* gene (GenBank MT084761). We interpret these findings as a result of evolutionary intron loss. Intron loss can be a sign for pseudogenization [46], meaning that the respective gene is not transcribed into mRNA any more, which normally serves as a template for translation into the respective protein.

Further sequence features of pseudogenization can be mutations at the exon/intron splice sites, the presence of non-synonymous mutations like the replacement of cysteine residues, an abundance of aromatic amino acids, or the appearance of premature stop codons, as described by Moleirinho et al. (2011) to identify human *MT* pseudogenes [46]. However, both md-MTs of *A. biplicata* possess characteristic cysteine positions and lack premature stop codons. Apart from that gastropod, MTs may be an exception to the rule that the introduction of aromatic amino acids will lead to *MT* pseudogenes. In fact, MTs of several gastropod species like *Cornu aspersum*, *Littorina littorea,* or *Pomatias elegans* contain aromatic amino acids in their peptide sequences but the respective MT proteins are still functional in vivo [6,26]. All these features argue against the pseudogenization of the two intronless *MT* genes. Overall, the internal architecture of *MT* genes can be very variable (Figure 3), and three different kinds of *MT* gene structures can be distinguished: (i) a typical exon/intron structure as known for many other genes, too [13,47,48,49,50]; (ii) a deviated exon/intron structure with a large exon consisting of repeat sequences that sometimes represent multiple domains [47]; and (iii) real intronless genes where introns are not present anymore [51] (this study). In addition, the length of the introns can vary to a great extent, mostly in contrast to the rather uniform size of exons (Figure 3). So far, the only further example of an intronless *MT* gene structure was reported for the ciliate *Tetrahymena pyriformis* [51,52] (Figure 3). Apart from the MTs mentioned above, there are other examples of intronless genes that can be activated to yield functional mRNA transcripts, too [53,54]. In humans, most of these genes encode proteins that are involved in signal transduction, growth, proliferation, or development [55,56]. It was shown that intronless genes evolve more rapidly than spliced genes [57]. This may be a possible advantage to adapt faster to changing habitat conditions, and may confer to the two intronless *md-MT* genes of *A. biplicata* a high evolutionary potential for adaption to fluctuating environmental metal levels on an evolutionary time scale. In the case of *Drosophila melanogaster*, for example, DNA-mediated duplication of entire *MT* genes correlate with the Cd resistance of this species at a population-specific level [29,58].

**Figure 4:**
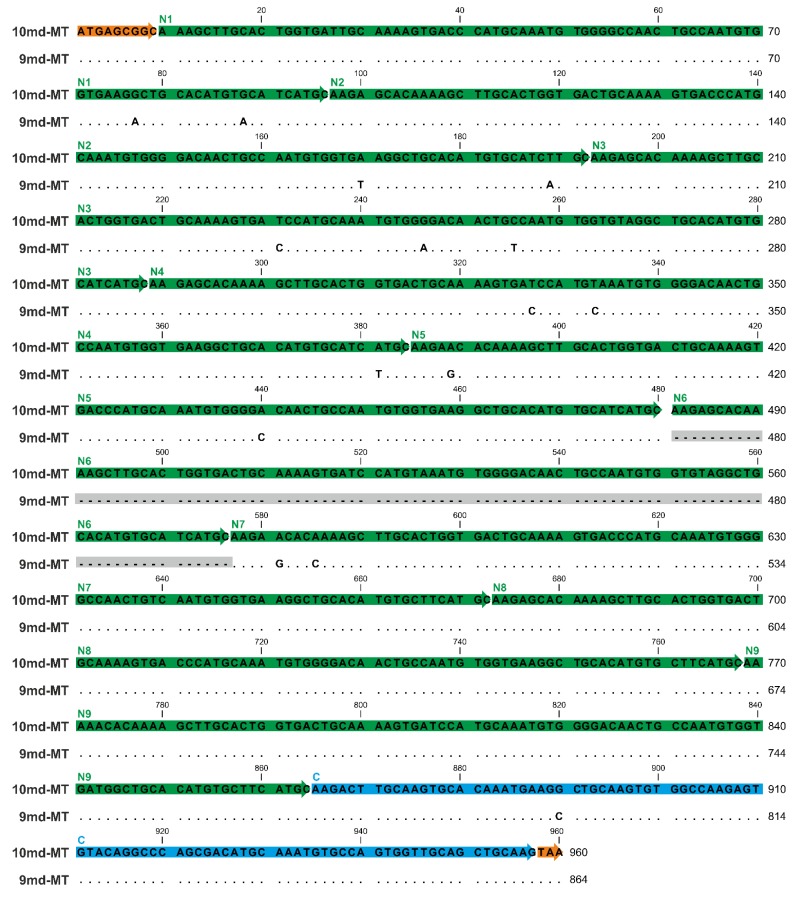
Gene/RNA sequence alignment of the two *md-MT* isoform genes (*10md-MT* and *9md-MT*) of *A. biplicata*. The gene sequences correspond to the detected RNA sequences because introns are lacking in the two *9md* and *10md*-*MT* genes. The orange boxes indicate the respective start sequences and the stop codon. The N-terminal repetition domains (N1–N9) are highlighted through green arrows, whereas the C-terminal domain is marked in blue. Dots represent identical nucleotides present in both *md-MT* genes; base replacements between the two genes are indicated by respective letters. The missing sequence of the N6 domain within the *9md-MT* gene is shadowed in grey.

Furthermore, both *md-MT* genes of *A. biplicata* are transcribed to RNA (Figure 4) [19]. Hence, due to the lack of introns, the DNA sequence of both *md-MTs* matches its respective RNA transcripts (Figure 4). The shorter *9md-MT* gene has a length of 864 nucleotides encoding for nine metal-binding domains, consisting of 8 repetitions (N2-8) of a primordial N-terminal domain (N1), in addition to a distinctly different C-terminal domain (C1). The second *10md-MT* gene spans a length of 960 nucleotides that translate into an MT with 10 metal-binding domains, 9 of which (N2-9) represent repetitions of the N-terminal domain (N1), in addition to a different C-terminal domain (C1) (Figure 4). The final arrangement of the respective N and C-domains was achieved by comparing the resulting putative md-MT peptide sequences with already known gastropod MT structures [25,26] (for detailed discussion, see Section 2.6).

To compare the single domains with each other, a BlastN (high similarity, default parameters, NCBI homepage) was performed (see Table S2). While the respective C-terminal domains of the two md-MTs are, with a sequence similarity of 98.92%, highly similar among each other, no similarity can be observed between them and the repetitive N-terminal domains. On the other hand, the identity or high homology among all N-terminal domains are evident, irrespective of whether they belong to the *9md-MT* or *10md-MT* isoform. Yet, a comparison between *9md-MT* and *10md-MT* reveals that some of the N-terminal repetition domains are identical even across the two isoforms (Figure 4, Table S2).

For example, the domains N4 of *9md-MT* and N2 of *10md-MT* share a sequence similarity of 100%. A ranking of N-terminal repeat domains according to their sequence similarity from highest to lowest homology in the two *md-MT* isoforms shows the following patterns:

*9md-MT*: N2, N4, N5, N8 > N3, N9 > N7 > N1.
*10md-MT*: N3, N4, N6 > N5, N8, N2 > N9 > N7 > N1;

In both isoforms, the domains N1 and N7 are the most divergent sequences compared to all other N-domains within the respective *md-MT* gene. In the case of N1, the aberrance can be explained by the shorter length of this domain due to a missing linker region in the front (Figure 4). Overall, the structural organization of the md-MTs of *A. biplicata* is closely related to that of the three-domain MTs of *Littorina littorea* and *Pomatias elegans* [26,27], which can both be regarded as md-MTs with only one repetition of the N-terminal metal-binding domain. Apart from that, it has also been demonstrated that the C-terminal metal binding domains are highly conserved among all snail MTs. At least in the three-domain MT of *Littorina littorea*, the C-terminal domain has assumed an important role in cooperative uploading of Cd^2+^ to the whole MT protein [19].

A possibility for the emergence of these *md-MT* intronless genes in *A. biplicata* could be RNA-based gene duplication by retroposition. Typical features of such retrocopies (retrogenes and retropseudogenes) are, besides the lack of introns, a spatial distribution within the genome distant from the parent genes, target site duplications, and a poly-A tail [59]. Among MTs, one example of a retropseudogene that is not transcribed into RNA is the human MTII_B_ [60]. In contrast, some transgenes are only transcribed into RNA and can play a regulatory role [61], whereas other transgenes are transcribed to mRNAs that encode for full-length proteins [62]. Alternatively, the two *md-MT* genes of *A. biplicata* may have originated by duplication of a primordial *MT* gene, followed by independent duplication of the N-terminal domains in the two genes. The original introns of the two *md-MT* genes may thereafter have been lost over time. Several studies have shown, in fact, that intron loss is much more likely to occur than intron gain [63,64]. Another scenario would be the emergence of one *md-MT* gene by the process described above and a subsequent duplication of the whole *md-MT* gene, thereby losing or gaining additional N-terminal domains. Further studies will have to clarify by which kind of duplication events the *md-MT* genes of *A. biplicata* may have originated, and if the respective mRNAs are translated into full-length md-MT proteins.

### 2.4. Impact of Cd Exposure on MT Gene Transcription in Alinda biplicata

In order to explore whether and to which degree Cd accumulation in *A. biplicata* might be linked to MT expression, reverse transcription of *md-MT* genes was optimized and qRT PCR was performed. Sequence confirmation of *md-MT* mRNA transcripts appeared to be challenging. Upon blasting of the cDNA sequences of the CuMT of *A. biplicata* (GenBank: MK639793) or other known CdMTs against transcriptomic data (SRX 7671047), it appeared that the respective contigs consisted only of partial sequences of the full-length *md-MT* transcript (see Figure S2). A possible reason for this could be the repetitive nature of the *md-MT* RNA sequences [65] or the error proneness of the reverse transcription process of RNA into cDNA [66,67]. In a comparative approach, three different transcriptase systems, namely, the RevertAid H Minus (RA), SuperScript^TM^ IV (SSIV) and AccuScript High Fidelity (AS), were tested (see Material and Methods). Upon PCR using cDNA-templates from all three tested reverse transcriptases, there appeared some additional bands on the gels that were not shared by all three approaches. A reason for this could be an intramolecular template switching of the respective reverse transcriptases during cDNA synthesis. In this case, the RT enzyme switches within the template from one repeat to another, which could be enhanced by secondary structures of the respective RNA template [66,68]. Only the approach with the AccuScript High Fidelity kit produced one single amplification band. Hence, we decided to use for our study the AccuScript High Fidelity kit (Agilent Technologies Inc., Santa Clara, CA, USA) to generate cDNAs for further downstream analysis by qRT-PCR.

**Figure 5:**
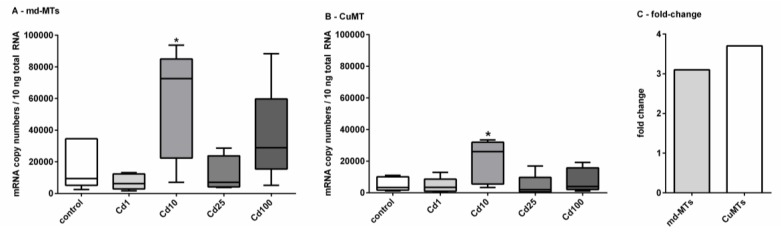
Impact of Cd exposure on *MT* gene transcription in *A. biplicata*. Whisker box plots for mRNA copy numbers are given for (**A**), showing the joined transcriptional upregulation of the two *md-MT* genes (*9md-MT* + *10md-MT*) and (**B**), of the *CuMT* gene in controls and Cd-treated snails (*n* = 5 each) from the lowest to the highest value of each treatment group. The respective box extends from the 25th to the 75th percentile, with the square line showing the median. (**C**), Bar graph reflecting the fold-change of joined *md-MT* and *CuMT* transcription in snails treated with 355.3 µg/g Cd (Cd10), referred to control levels. Cd exposure concentrations were as follows: Cd1 = 38; Cd10 = 355.3; Cd25 = 804.5; Cd100 = 2692.6 µg/g d.w. (see Section 3.2). Asterisks (*) indicate significance (*p* < 0.05) of mRNA copy numbers referred to respective control values.

*MT* gene expression of *the md-MTs* and the two-domain *CuMT* of controls and Cd-treated snails were quantified via qRT-PCR. It is important to stress that in the case of the two *md-MT* genes, the quantitative RNA measurements did not distinguish between the *9md-MT* and *10md-MT* isoforms (see Material and Methods for further explanation). In contrast to the increasing Cd concentrations in the snail tissues in a dose-dependent manner up to a threshold level (Figure 1), the influence of Cd exposure on *MT* gene transcription of *A. biplicata* was more variable (Figure 5).

A significant upregulation for the *md-MT* genes and the *CuMT* gene was only detected in snails fed on cookies with a Cd concentration of 355.3 µg/g d.w. (Cd10). In this case, a 3-fold induction for the *md-MT* genes and a 4-fold induction for the *CuMT* gene were observed (Figure 5C) There might be several reasons for this variable response of *MT* gene transcription in Cd-exposed *A. biplicata*. For example, under control and low-dose Cd exposure (Cd1) conditions, sufficient MT protein might already be available to bind the excess amounts of Cd and hence, no transcriptional upregulation of the respective *MT* genes would be necessary. In some non-exposed helicid snails, the *CdMT* gene is transcribed, but the respective encoded MT protein is not expressed [22]. Inhibition of mRNA translation and its activation upon stress is a phenomenon described for many different genes [69,70,71]. In *E. coli*, for example, the enzyme Aconitase B is bound to the mRNA of superoxide dismutase (SOD) and inhibits its translation under control conditions. Under oxidative stress, however, the translational inhibition by this enzyme is reversed, and so the SOD protein can be synthesized [70].

At moderately increased Cd exposure concentrations in our study (Cd10 in Figure 5), *MT* genes (md-MTs and *CuMT*) of *A. biplicata* are upregulated with an approximately 3.5-fold induction (Figure 5C), very probably in response to Cd stress in order to handle its detoxification. Such a rather moderate activation for *MT* gene expression due to Cd exposure was reported for other gastropod species, too, such as *Helix pomatia* or *Cornu aspersum* [14,19], where Cd^2+^ ions are readily detoxified by binding to the produced CdMT proteins [6,72]. In contrast, Cd-dependent *MT* gene upregulation is much stronger in vertebrates like rats [73], fish [74,75,76], or other invertebrate species like in the earthworm *Eisenia fetida* [77]. When Cd exposure levels exceed a certain threshold concentration, *MT* gene transcription in *A. biplicata* is not upregulated anymore, or it may even be inhibited (Figure 5). One reason for this may be that Cd uptake by *A. biplicata* up to such high levels may lead to an exhaustion of its MT detoxification system. As a consequence, metal toxicity to the snail may increase to an excessive level as to inhibit the protein expression machinery. A strong indication for this may be the feeding avoidance behavior and inactivity of snails observed at Cd concentrations above a threshold level of 355.3 µg Cd/g (Cd10) in the food cookies (Figure 2).

### 2.5. Are Alinda biplicata md-MTs Involved in Cd Detoxification?

**Figure 6:**
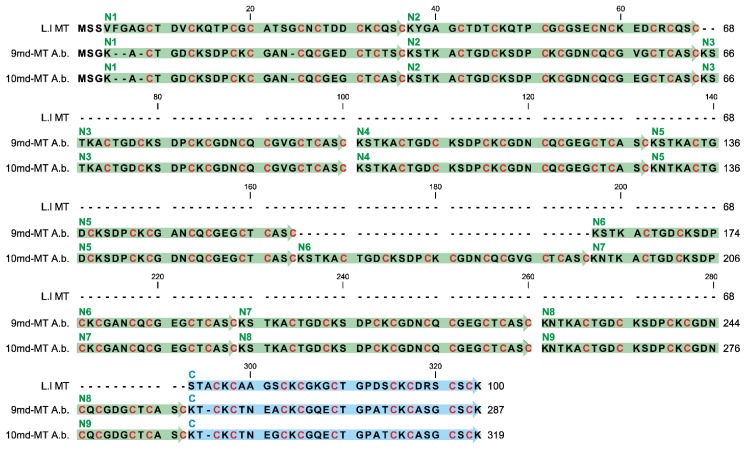
Alignment of putative multi-domain MT proteins from *A. biplicata* with the previously published 3md-MT from *Littorina littorea* [26,28]. N-terminal domain sequences (N1–N9) are highlighted in green, whereas the C-terminal domains are colored in blue. Cysteine residues are highlighted with red letters for C.

The *10md-MT* gene of *A. biplicata* encodes a putative md-MT protein with 319 aas, thus being the longest MT ever so far described from a mollusk species. Only for the sea squirt *Oikopleura dioica* a longer MT variant with 399 aas was reported [47]. In addition to the *10md-MT*, *A. biplicata* possesses a second large-sized md-MT isoform with 287 aas, encoded by the *9md-MT* gene (Figure 4 and Figure 6).

A multiple alignment reveals that across all N-terminal repetition domains of 9md-MT and 10md-MT, only two variable amino acid positions are present (Figure S3A). Hence, a consensus sequence for the N-terminal domains from both md-MTs of *A. biplicata* can be assessed as follows: CTGDCKSDPCKCG***X_1_***NCQCG***X_2_***GCTCASC. In this sequence, alanine (A) or aspartic acid (D) can replace each other at position X_1_, whereas at position X_2_, glutamic acid (E) can occur additionally to A and D (Figure S3A). One exception of this is seen in the N1-domain of 9md-MT, which contains two additional amino acid replacements (G → D; A → T; Figure S3A).

A comparison of amino acid sequences of these large-sized md-MTs with the three-domain MT of *Littorina littorea* shows the high degree of similarity between the respective N-terminal domains and their tandem repeats on the one hand, and the respective C-terminal domains on the other (Figure 6).

Since the three-domain MT of *Littorina littorea* clearly exhibits Cd-selective binding features [28] that were confirmed by solution NMR [26], it is suggested that, based on the high degree of sequence similarity, the two md-MTs of *A. biplicata* might also be Cd-specific. It also appears that both md-MTs are more closely related to other snail CdMTs, including those of *Helix pomatia* and *Cornu aspersum* (61–69% identity), than to the respective aminoacid sequence of snail CuMT isoforms (48–54% identity) (Figure S3B) [19]. All three MT sequences shown in Figure 6 share, in close vicinity to Cys residues, an elevated frequency of particular amino acids that were discussed to play a potential role in promoting Cd-selective binding features of the respective MT proteins (Table 1).

Namely, a high preponderance of lysine (K) over asparagine (N) residues, as observed in the two md-MTs of *A. biplicata*, was considered to be a typical feature of Cd-selective snail MTs (Table 1) [24,78]. In the MT of *Biomphalaria glabrata,* for example, the replacement of a lysine by an asparagine residue increased the Cu but not the Cd-binding capability of the respective MT protein [79]. In addition, Cd-selective snail MTs typically exhibit a higher content of charged amino acids, compared to non-metal-specific variants [24] (Table 1). This may contribute to a tighter protein folding caused by increased electrostatic interactions [24,80]. From a biological point of view, a stiffer protein folding may help to prevent the loss of already bound Cd^2+^ ions, which would otherwise cause intracellular damage upon release. Further, the occurrence of histidine (H), which is frequently observed in snail CuMTs, is missing in the two md-MTs of *A. biplicata* (Figure 6, Table 1). Even though this amino acid normally decreases the Cu-binding ability of MTs, it seems to play a vital role in these proteins for an optimal balance between Cu binding and Cu release, in order to fulfill its biological role in Cu homeostasis [81,82].

**Figure 7:**
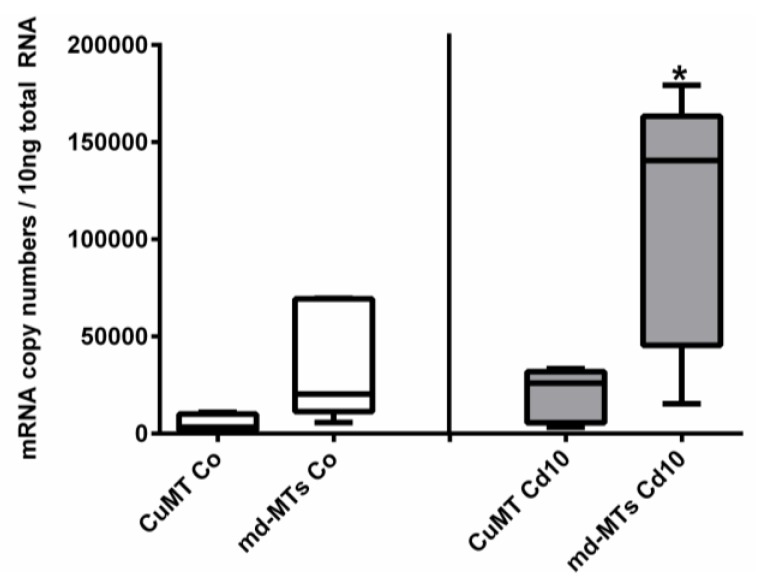
Comparison of mRNA expression levels of *CuMT* versus joined *md-MT* genes (*9md-MT* + *10md-MT*) in *A. biplicata*. Whisker box plots from the lowest to the highest values, showing transcription levels for controls (white whisker box plots, left-hand side) and snails exposed to 355 µg/g d.w. (Cd10) (grey whisker box plots, right-hand side; *n* = 5 each). The box of each treatment group extends from the 25th to the 75th percentile, with the square line showing the median. Asterisks (*) indicate significance (*p* < 0.05) upon comparison between *CuMT* transcription versus *md-MT* transcription in exposed snails (Cd10).

In addition to all these primary structure features, the *md-MT* genes of *A. biplicata* exhibit a transcription pattern that is similar to other Cd-specific snail MTs [14,22]. Seemingly higher basal *md-MT* gene transcription levels in unexposed individuals, and significantly higher gene transcription values in Cd-treated *A. biplicata* snails, compared to the respective *CuMT* gene, strongly suggest an elevated expression rate and Cd-dependent activation of the *md-MT* genes of this species and hence, their involvement in Cd inactivation (Figure 7). This is in accordance with the transcriptional expression patterns of the *CdMT* genes in the helicid snail species *Helix pomatia* and *Cornu aspersum* [22]. A minor different transcription pattern is observed for the *CuMT* gene of *A. biplicata,* which, contrary to *CuMT* genes of other snails [22,83], is slightly upregulated by Cd exposure, too (Figure 5 and Figure 7). This indicates that the *CuMT* gene of *A. biplicata* may behave more like an unspecific Cd/CuMT rather than a true CuMT [6,84].

### 2.6. Structural and Evolutionary Considerations of the Emergence of md-MTs in Alinda biplicata

**Figure 8:**
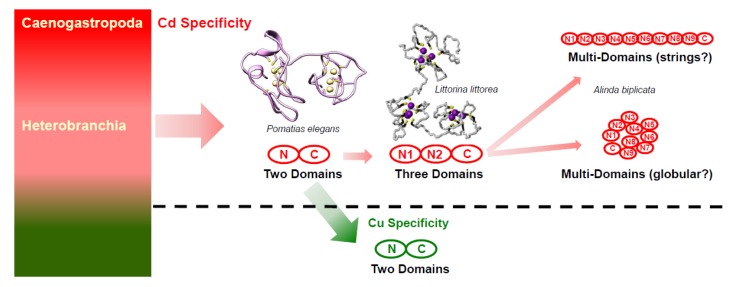
Hypothesis of evolution of metal-specificity and structure in MTs of the two gastropod clades Heterobranchia and Caenogastropoda (left-hand, multi-colored box). Two-domain or multi-domain Cd-specific MTs (CdMTs) in Caneogastropoda and Heterobranchia are shadowed in pink. Two-domain Cu-specific MTs (CuMTs) in some lineages of Heterobranchia are shadowed in green. Adapted from Dallinger 2018 [86].

An additional enhancement of the Cd-inactivating capacity of 9md-MT and 10md-MT may arise from their multi-domain structure, which strongly suggests an increased metal-binding capacity of the two md-MTs of *A. biplicata* due to their correspondingly higher Cd^2+^ binding stoichiometry. In other snail species, for example, the Cd-binding stoichiometry increases from 6 to 9 Cd^2+^ ions bound per protein molecule if an additional metal-binding domain is added to the protein through evolutionary optimization. This was experimentally demonstrated by NMR or mass spectrometry for the Cd-specific MT of *Littorina littorea* [26,28] (Figure 8). Interestingly, in Cu-specific MTs of fungi such as *Tremella mesenterica* and *Crypotococcus neoformans*, too, the addition of Cys-containing tandem repetitions increases their Cu-binding capacity [49,78,85].

If the binding stoichiometry of 6 Cd^2+^ ions per domain is extrapolated from so-far known two- or three-domain snail CdMTs [23,26] to the md-MTs of *A. biplicata*, we would expect a binding stoichiometry of 27 Cd^2+^ ions for the 9md-MT, and of 30 Cd^2+^ for the 10md-MT, respectively. Consequently, this would attribute to the two md-MTs of *A. biplicata*, the highest Cd-binding capacity ever observed in any MT molecule. How such long md-MTs are organized in their tertiary architecture still remains unclear. One possibility would be to assume a globular structure or, as an alternative hypothesis, the domains could also be organized as strings (Figure 8). This is relevant because the three-dimensional structure has implications on the degree of reciprocal contacts of the single domains to each other, which in turn might influence their metal loading capacity, as shown in the three-domain MT of *Littorina littorea* [19]. Further studies will be needed to elucidate the tertiary organization and Cd binding capability of such long md-MTs in snails.

## 3. Material and Methods

### 3.1. Animal Rearing and Acclimatization

Individuals of *Alinda biplicata* (A.b.) were collected in summer 2017 and 2018 from mural structures in the vicinity of the department of zoology (Technikerstraße 25, A-6020 Innsbruck) of the University of Innsbruck, Austria. Snails were acclimatized in transparent octagonal plastic boxes (diameter: 12 cm; height: 6 cm) without substrate and water to keep them inactive under stable laboratory conditions (12 h dark/12 h light and temperature 20 °C) for one to two weeks. Subsequently, snails were divided into six experimental groups with 10 adult snails each, and five snails per treatment group were put into petri dishes (92 × 16 mm) (Sarstedt, Nümbrecht, Germany). Adult individuals were identified by the number of whorls (minimum 10) and the formation of the lip and closing apparatus [2].

### 3.2. Cd Exposure and Tissue Dissection

The control group was supplied with uncontaminated snail cookies (1 µg Cd/g d.w.), whereas Cd-treated snails were fed on Cd-containing snail cookies through four days. Snail cookies were produced as follows: 2 g Agar Kobe-Kobe I (Roth, Karlsruhe, Germany), 0.66 g CaCO_3_ (Merck Chemicals, Vienna, Austria) and 0.25 g algae flakes Novo Malawi (JBL GmbH Co KG, Neuhofen, Germany) were dissolved in 85 mL deionized water and heated up in the microwave for approx. 30 s. After cooling down the mixture, following amounts of a 1% CdCl_2_ solution (Sigma Aldrich by Merck, Darmstadt, Germany) were added: 0.01 mL (Cd1), 0.1 mL (Cd10), 0.25 mL (Cd25), 1 mL (Cd100), and 2 mL (Cd200). The subsequently measured Cd concentrations (µg/g dry weight) were 1 (controls), 38 (Cd1), 355.3 (Cd10), 804.5 (Cd25), 2692.6 (Cd100), and 6396.1 (Cd200), respectively. For exposure, cookies were placed in the middle of lidded plastic petri dishes (Saarstedt, Nümbrecht, Germany) along with 5 individual snails per petri dish. During exposure, small water drops were put into the petri dishes and on snail cookies every day to prevent desiccation. After four days of Cd exposure, individuals were sacrificed on an ice-cooled aluminum plate intermittently cleaned with RNase AWAY^®^ (Sigma Aldrich by Merck, Darmstadt, Germany) and 70% ethanol. Multi-tissue aliquots from five individuals of each experimental group were stored separately in RNAlater^®^ (Fisher Scientific, Vienna, Austria) at −80 °C for RNA isolation. The remaining parts of soft tissues were collected for subsequent measurement of Cd tissue concentrations (*n* = 5 per experimental group).

For DNA isolation, additional snails were collected from the same site and sacrificed as described before. Multi-tissue parts of three adult individuals were pooled to one sample and stored in RNAlater^®^ (Fisher Scientific, Vienna, Austria) at −80 °C for further processing.

### 3.3. Cd Analysis

Sample digestion and analysis of Cd tissue concentrations were performed as described previously [27]. Shortly, a 1:1 mixture of nitric acid (65%) (Suprapur, Merck, Darmstadt, Germany) and deionized water were pipetted into lidded Eppendorf tubes containing the oven-dried samples (multi-tissue samples, snail cookies, and reference material) and digested under pressure in a heated (70 °C) aluminum block. Cd concentrations were measured by furnace atomic absorption spectrophotometry (Z-8200 Polarized Zeeman atomic absorption spectrophotometer with SSC-300 auto sampler; Hitachi, Üdern, Germany). The system was calibrated with diluted Cd solutions from a 1000 ppm Titrisol Cd Standard (Merck, Darmstadt, Germany). TORT-2 lobster hepatopancreas (NRC, Ottawa, Canada) was used as a certified standard reference material. The mean concentration value of the measured standard reference material (27.24 µg/g d.w.) was within the specified range.

The bioaccumulation factor (BAF) was calculated by dividing the mean of Cd tissue concentrations of the respective Cd exposure group by the mean of Cd tissue concentrations of controls. For calculation of the bioconcentration factor (BCF), the mean of Cd tissue concentrations of controls was first subtracted from the mean Cd tissue concentrations of every Cd-exposure group. Subsequently, the respective mean value of Cd treatment groups was divided by the measured Cd concentration of the respective snail cookie.

### 3.4. DNA Isolation and Long Distance (LP)-PCR of md-MT Genes

All samples were homogenized with a Precellys^®^ homogenizer (Bertin Instruments, Montigny-le-Bretonneux, France). Genomic DNA was obtained using the DNeasy Plant Mini Kit (Qiagen, Hilden, Germany) and quality was assessed visually by agarose gel electrophoresis. In order to obtain longer fragments upon amplification of the *md-MT* genes, the Platinum^TM^ SupferFi^TM^ Green PCR Master Mix (Invitrogen, Thermo Fisher Scientific, Waltham, MA, USA) was applied for PCR amplification (for primers and PCR setup, see Table S2). PCR products were separated on a 1.5% agarose gel (Biozym, Hessisch Oldendorf, Germany) by electrophoresis. Gene-specific bands were excised, purified with the QIAquick™ Gel Extraction Kit (Qiagen, Hilden, Germany) and cloned with the TOPO™ XL-2 Complete PCR Cloning Kit (Invitrogen, Thermo Fisher Scientific, Waltham, MA, USA) for Sequencing (Invitrogen, Thermo Fisher Scientific, Waltham, MA, USA). Insert-containing plasmids were purified using the QIAprep Spin Miniprep Kit (Qiagen, Hilden, Germany) and sent to Microysnth AG (Balgach, Switzerland) for Sanger sequencing.

### 3.5. RNA Isolation, Quantification and cDNA Synthesis

**Figure 9:**
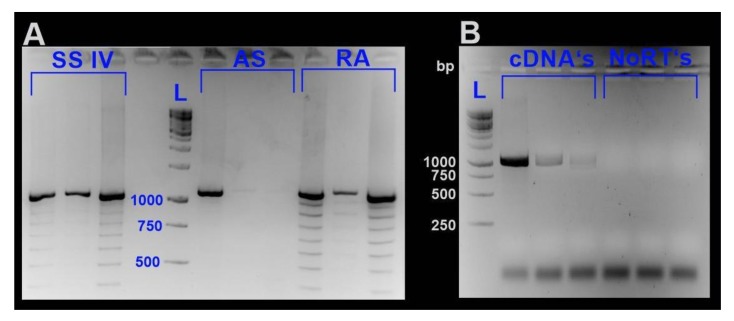
Gene-specific LD-PCR of the *md-MTs* using cDNA templates generated by three different reverse transcriptase systems. (**A**) PCR products using cDNAs as a template, which were generated by three different reverse transcriptases, namely, SuperScript^TM^ IV (SSIV), AccuScript High Fidelity (AS), and RevertAid H Minus (RA). (**B**) Shows PCR products using cDNAs generated by reverse transcription with AccuScript High Fidelity. In addition, the respective NoRT’s (see Section 3.6) are shown as a negative control to exclude contamination of the cDNAs with genomic DNA. Abbreviations: L., ladder.

All samples were homogenized with a Precellys^®^ homogenizer (Bertin Instruments, Montigny-le-Bretonneux, France). Total RNA was isolated by applying the RNeasy Plant Mini Kit (Qiagen, Hilden, Germany), including a DNase I digestion step according to the manufacturer’s instruction. RNA integrity was checked by agarose gel electrophoresis. RNA concentrations were estimated applying the Quant-iT™ RiboGreen^®^ RNA Assay Kit (Life Technologies Corporation, Carlsbad, CA, USA) with the Victor™ X4 2030 Multilabel Reader (Perkin Elmer, Waltham, MA, USA). To minimize the probability of generating artificial sequences, we decided to test three different transcriptase systems to optimize cDNA synthesis for further downstream analyses. For confirmation of *md-MT* mRNA sequences [19], cDNAs were generated applying three different reverse transcriptase systems, namely, RevertAid H Minus Reverse Transcriptase (Thermo Fisher Scientific, Waltham, MA, USA), SuperScript^TM^IV Reverse Transcriptase (Thermo Fisher Scientific, Waltham, MA, USA), and AccuScript High Fidelity Reverse Transcriptase (Agilent Technologies Inc., Santa Clara, CA, USA) according to the instructions of the manufacturers. All three reverse transcriptases are RNase H-deficient enzymes derived from the Moloney murine leukemia virus (MMLV). After generating cDNAs, as stated in the respective protocol of each enzyme, LD PCR was performed with gene-specific primers (see Table S2) applying the Advantage^®^ 2 PCR Kit (Clontech, Takara Bio Europe, Saint-Germain-en-Laye, France). When analyzing PCR products using cDNAs generated with RA and SSIV as a template, multiple bands were abundant. In contrast, amplification products derived from LD PCR with cDNA-templates generated by AS clearly showed less visible bands (Figure 9). However, PCR products of all cDNAs possessed the most prominent band with a length of approximately 1000 bp (Figure 9).

As shown by Cocquet et al. [66], reverse transcriptases with an enhanced thermostability can lead to overcoming the generation of artificial bands. However, this was not true in our case. The optimal working temperature of AS and RA it at 42 °C. SSIV has an even higher temperature optimum, and cDNA generation was performed at an incubation temperature at 55 °C, as recommended by the protocol. For sequence confirmation, the most prominent gene-specific bands shared by PCR products of all three reverse transcriptases were excised, purified with the QIAquick™ Gel Extraction Kit (Qiagen, Hilden, Germany) and sent to Microsynth for Sanger sequencing (Microsynth AG, Balgach, Switzerland). Due to sometimes low sequence quality of the contained sequences, cloning of clean PCR products was performed with the pCR™4-TOPO^®^vector of the TOPO^®^ TA Cloning^®^ Kit for Sequencing (Invitrogen, Thermo Fisher Scientific, Waltham, MA, USA). Insert-containing plasmids were purified using the QIAprep Spin Miniprep Kit (Qiagen, Hilden, Germany) and sent to Microysnth AG (Balgach, Switzerland) for Sanger sequencing. Consequently, we used AccuScript to generate cDNAs for further downstream analysis like qRT-PCR.

### 3.6. Quantification of MT Gene Transcription by Quantitative RealTime PCR (qRT-PCR)

For qRT PCR, 450 ng total RNA was used in a 20-µL approach for cDNA synthesis applying the AccuScript High Fidelity Reverse Transcriptase (Agilent Technologies Inc., Santa Clara, CA, USA). To exclude genomic contamination, NoRTs were generated treated in the same way as all other samples objected to cDNA synthesis, but without the addition of the reverse transcriptase. Gene-specific primers were designed with Primer Express 3.0 software (Applied Biosystems, Foster City, CA, USA) (see Table 2).

To avoid multiple binding to the highly conserved repetitive sequences of the N-domains within the respective *md-MT* gene and between both *md-MTs* (see Figure 4, Table S1), the C-domain was used for primer design. Consequently, the primer pair can bind only one time to one RNA transcript because the C-domain is present only once within the respective *md-MT* isoform (Figure 4). However, due to high sequence similarity of the C-domains between the *9md-MT* and *10dm-MT* sequences (see Figure 4), it was not possible to evaluate the RNA expression level of the two single *md-MT* isoforms independently. For optimization, a primer matrix was applied. The PCR products of the optimal primer concentrations (see Table 2) were obtained, cleaned, and cloned into a pCR™4-TOPO^®^vector, and resulting plasmids were sequenced (see detailed description above). Subsequently, calibration curves for both amplicons (Amplicon length: CuMT = 58 bp; md-MT = 89 bp) were established (CuMT: y = −3.4765x + 34.426; md-MT: y = −3.6511x + 38.629). To compare the expression levels of the two different amplicons, the PCR efficiency of the md-MTs was adjusted to 94%, as described in Pérez et al. (2013) [87]. Transcripts were quantified in a 10-µl approach using the Power SYBR^®^ Green PCR Master Mix (Applied Biosystems by Thermo Fisher Scientific, Waltham, MA, USA) with the QuantStudio^TM^ 3 (Applied Biosystems by Thermo Fisher Scientific, Waltham, MA, USA). The PCR amplification protocol was as followed: one initial denaturation step for 10 min at 95 °C followed by 40 cycles denaturation for 15 s at 95 °C and annealing/extension for 1 min at 60 °C.

### 3.7. Statistics

Data were analyzed and graphs were prepared using GraphPad Prism (Version 6.01) (GraphPad Software, San Diego, CA, USA). Significant outliers, proofed by the Grubb’s outlier test, were removed. Data were tested for normality using the Shapiro–Wilk normality test. All data were normally distributed. To analyze the different treatment groups, a Holm–Sidak multiple comparison test was applied. Significance level was set at *p* < 0.05. Sequences were analyzed and aligned in CLC Main Workbench 6.9 (Quiagen, Aarhus, Denmark), and the respective figures were refined in CorelDRAW X6 (Corel Corporation, Ottawa, ON, Canada). Gel images were taken with a Herolab Camera system using the E.A.S.Y Win32 program for visualization (Herolab, Wiesloch, Germany).

## 4. Conclusions

Cd accumulation and potential detoxification mechanisms were explored in the little terrestrial door snail, *A. biplicata*. Our data show that *A. biplicata* possesses great potential for Cd accumulation paired with a behavioral feeding avoidance strategy when exposed to excessively high Cd concentrations in the feed. The role of Cd-specific multidomain-MTs (9md-MT and 10md-MT) as potential candidates for Cd detoxification is discussed. In summary, our results contribute novel data to our knowledge on the metal handling strategy of *A. biplicata* and report for the first time the gene structure, mRNA expression, and amino acid protein sequence of such long md-MTs (domain > 3) in the gastropod species.

## Figures and Tables

**Table 1:** Different gastropod species MTs (left column) with abundance of amino acid residues (in single-letter codes) that are supposed to influence the metal-specific binding character of the respective MT proteins. The abundance of the selected amino acids refers to confirmed Cd- and Cu-specific gastropod MTs and snail md-MTs. The number (#) of selected amino acids (C, K, N, H) and the percentage (%) of charged amino acids (caas) in relation to the whole amino acid length (last column) of the respective MT peptide sequences (including the M at the start position) are listed. GenBank entry numbers are as follows: H.p. CdMT: AAK84863.1; C.a. CdMT: ABL73910.1; L.l. CdMT: AST14863.1; H.p. CuMT: AAK84864.1; C.a. CuMT: ABM55268.1, A.b. md-MTs (*9md-MT*: MK648139; *10md-MT*: MK648140).

MT	# of C	K/N Ratio	# of H	# caas	% caas	Length
*Helix pomatia* CdMT	18	9/1	0	16	23.90	67
*Cornu aspersum* CdMT	18	8/2	0	14	20.90	67
*Littorina littorea* CdMT	27	10/2	0	21	21	100
*Alinda biplicata* 9md-MT	81	36/10	0	66	23	287
*Alinda biplicata* 10md-MT	90	40/13	0	74	23.20	319
*Alinda biplicata* CuMT	17	6/6	1	11	16.90	65
*Helix pomatia* CuMT	18	5/6	1	12	18.50	65
*Cornu aspersum* CuMT	17	3/9	1	11	16.90	65

**Table 2:** Characterization of gene-specific primers used for MT mRNA quantification. Besides the sequence and length of the respective primer, also the primer efficiencies of both primer pairs estimated by generation of a standard curve are reported. Even though the primer efficiency of the second primer pair (*md-MTs*) is below 90%, this combination was used due to length limitation of the respective *md-MT* mRNA sequences.

Primer	Sequence 5′3′	Length [bp]	Conc. [nm]	Primer Efficiency
CuMT S	GCC TGC AAC AGC AAT CCA T	19	900	94%
CuMT AS	AAC AGG CAG CCC CAC ATT T	19	900	
md-MT S	GTG GTG ATG GCT GCA CAT GT	20	900	88%
md-MT AS	CGC TGG GCC TGT ACA CTC TT	20	900	

## Supplementary Materials

The following are available online at https://www.mdpi.com/1422-0067/21/5/1631/s1, Figure S1: Documented feeding behavior of controls and snails exposed to increased concentrations of Cd, Figure S2: Alignments of partial md-MT peptide sequences with the full-length *10md-MT*. Figure S3: (A) Alignmnent of all N-domains of the 9md-MT and 10md-MT of Alinda biplicata; (B) Sequence coverage of the 9md-MT and 10md-MT consensus sequences of Alinda biplicata (Ab) with Cd- and Cu-specific MTs of helicid snails Table S1: Sequence comparison off all single domains of the 9md-MT (9N1-9N9; 9C) and 10md-MT (10N1 – 10N9, 10C) of *Alinda biplicata*; Table S2: Characterization of gene specific primers and PCR parameters used for amplification of the *9md* and *10md-MT* mRNA.
