# Supplementary material for: Cadmium Uptake, MT Gene Activation and Structure of Large-Sized Multi-Domain Metallothioneins in the Terrestrial Door Snail Alinda biplicata (Gastropoda, Clausiliidae)
Source: Int J Mol Sci. 2020 Feb 27;21(5):1631. doi: 10.3390/ijms21051631 (PMC7084494; doi:10.3390/ijms21051631)
Supplement: Supplementary file 1 [file ijms-21-01631-s001.zip › ijms-726309-supplementary PROOF/Figure S1.pdf]

Figure S1: Documented feeding behaviour of controls (**A**) and snails exposed to increased concentrations of Cadmium (**B** - Cd1[38µg/g Cd]; **C** - Cd10[355.3µg/gCd]; **D** - Cd25[804.5µg/gCd]; **E** – Cd100[2692.6µg/gCd]; **F** – Cd200[6396.1µg/gCd]). Per treatement group two petridishes with five individuals each were used.

A - Controls

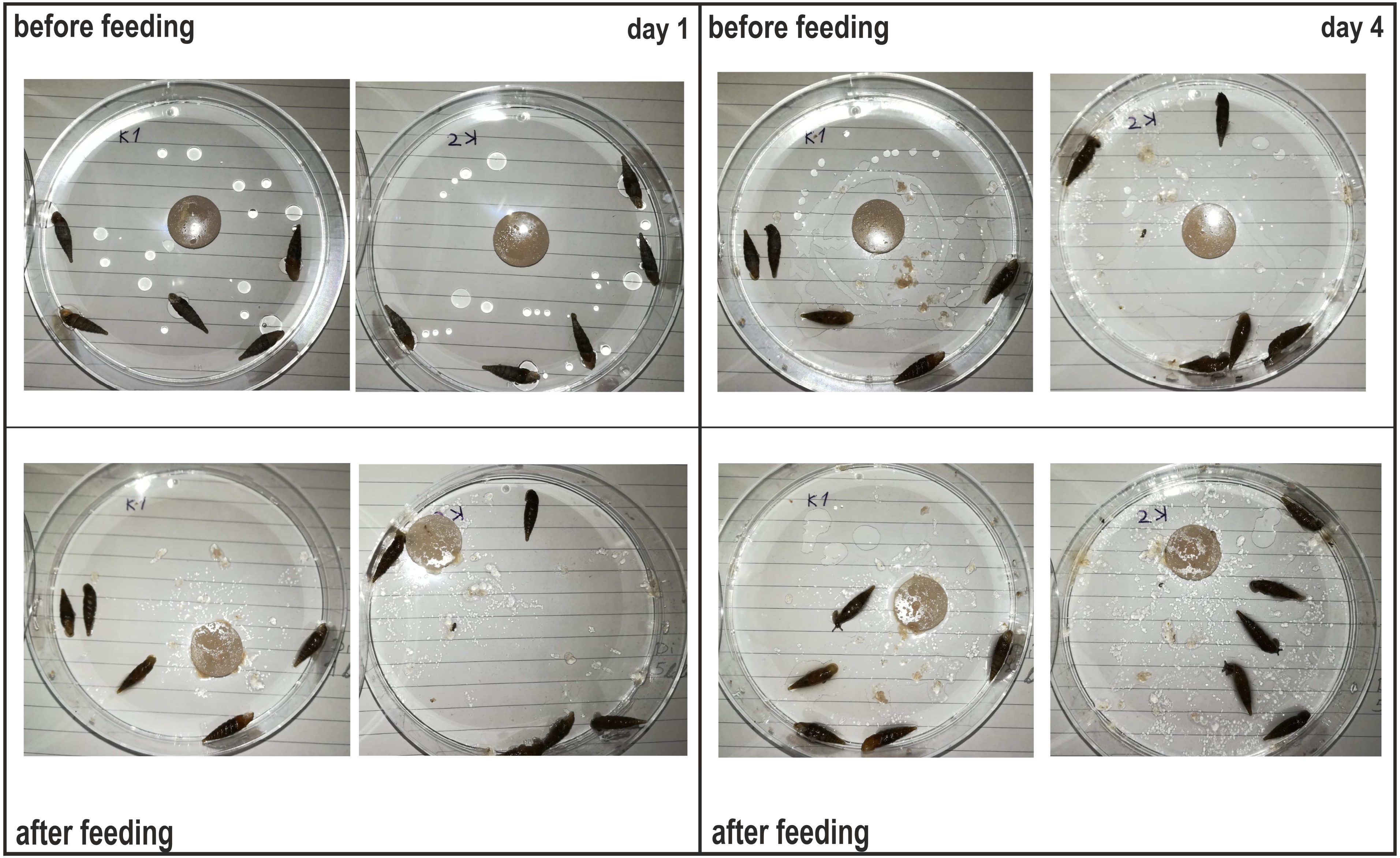

B - Cd1

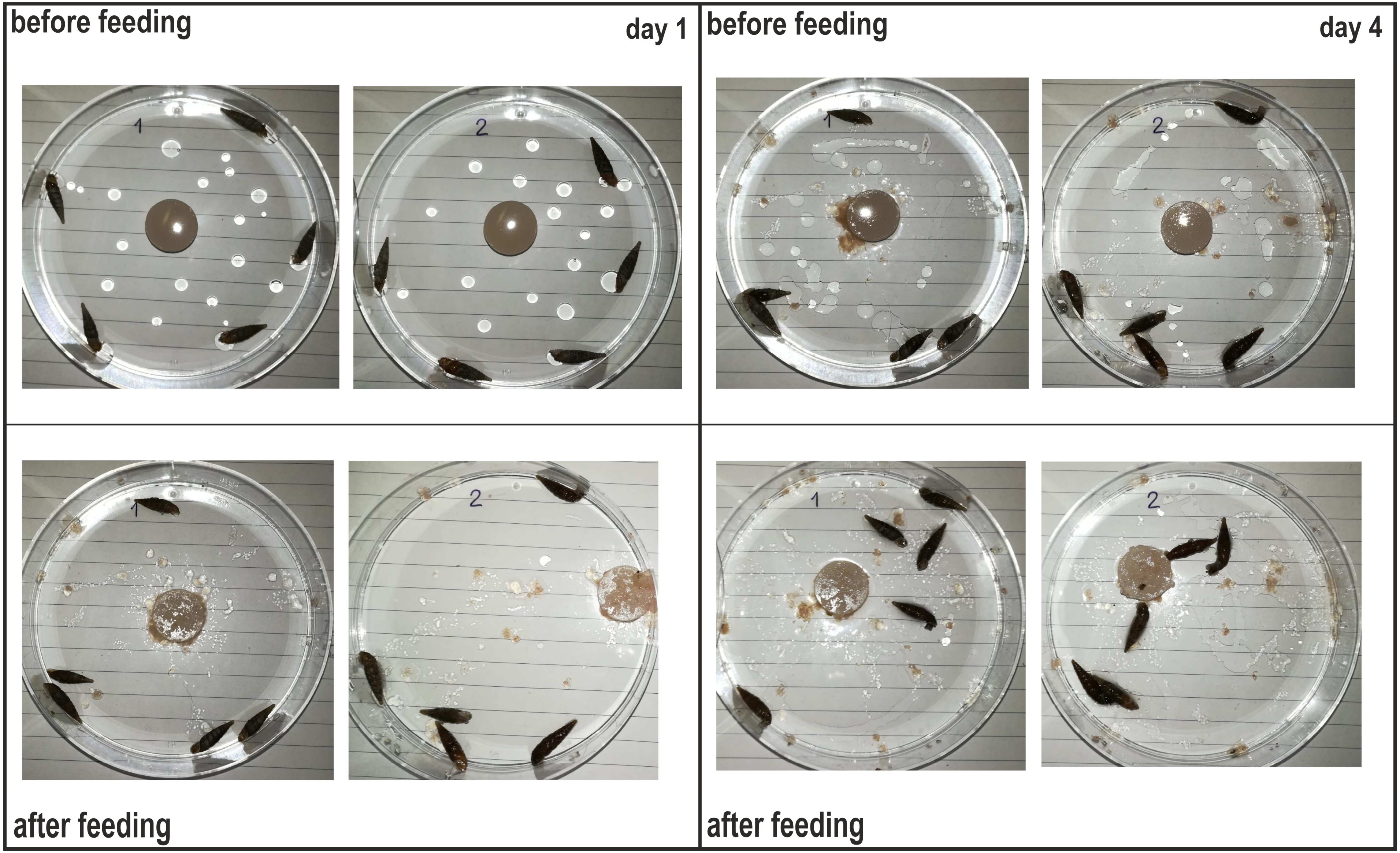
before feeding
day 1
before feeding
day 4
1
2
1
2
1
2
1
2
after feeding
after feeding

C - Cd10

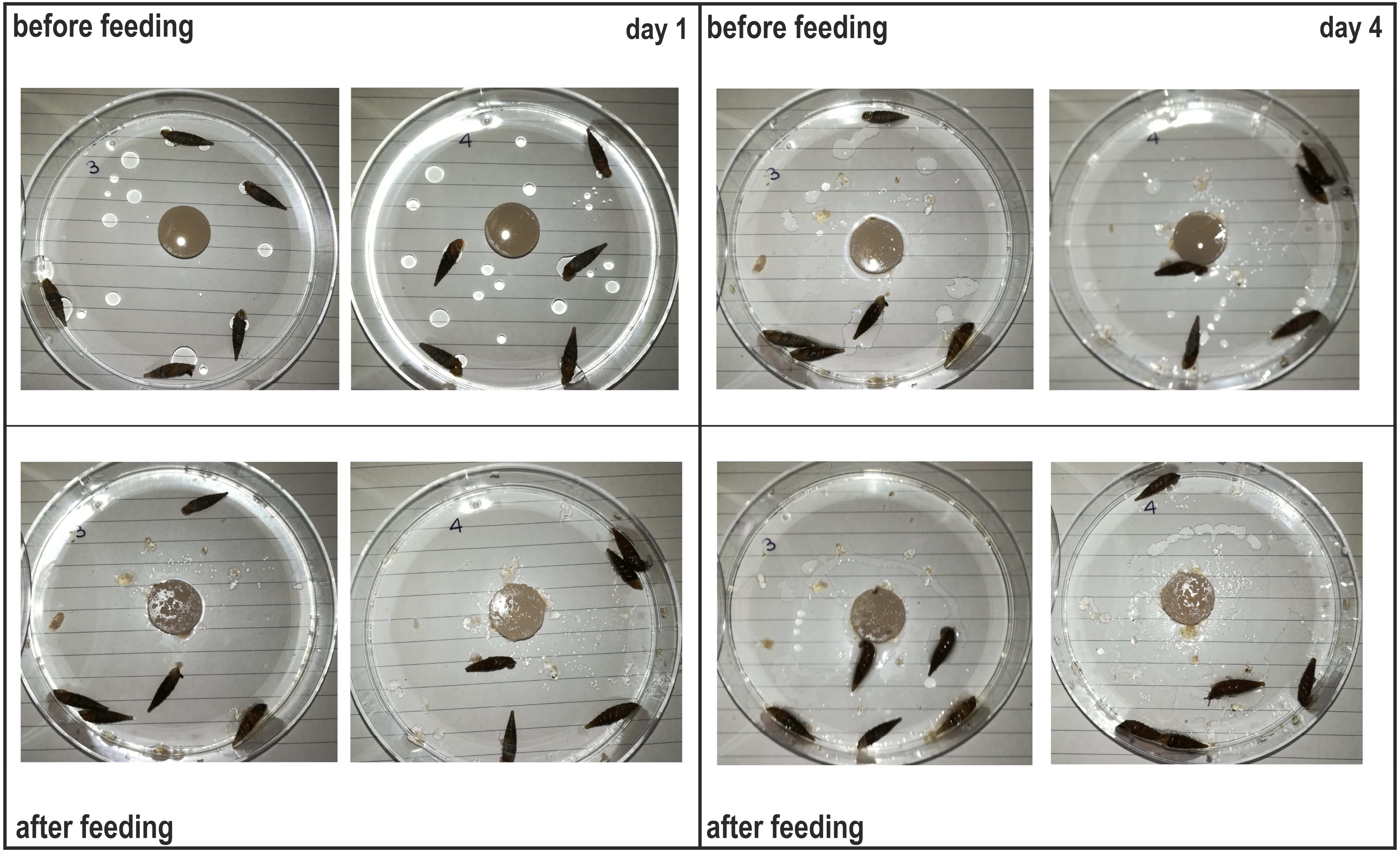
before feeding
day 1
before feeding
day 4
after feeding
after feeding

D - Cd25

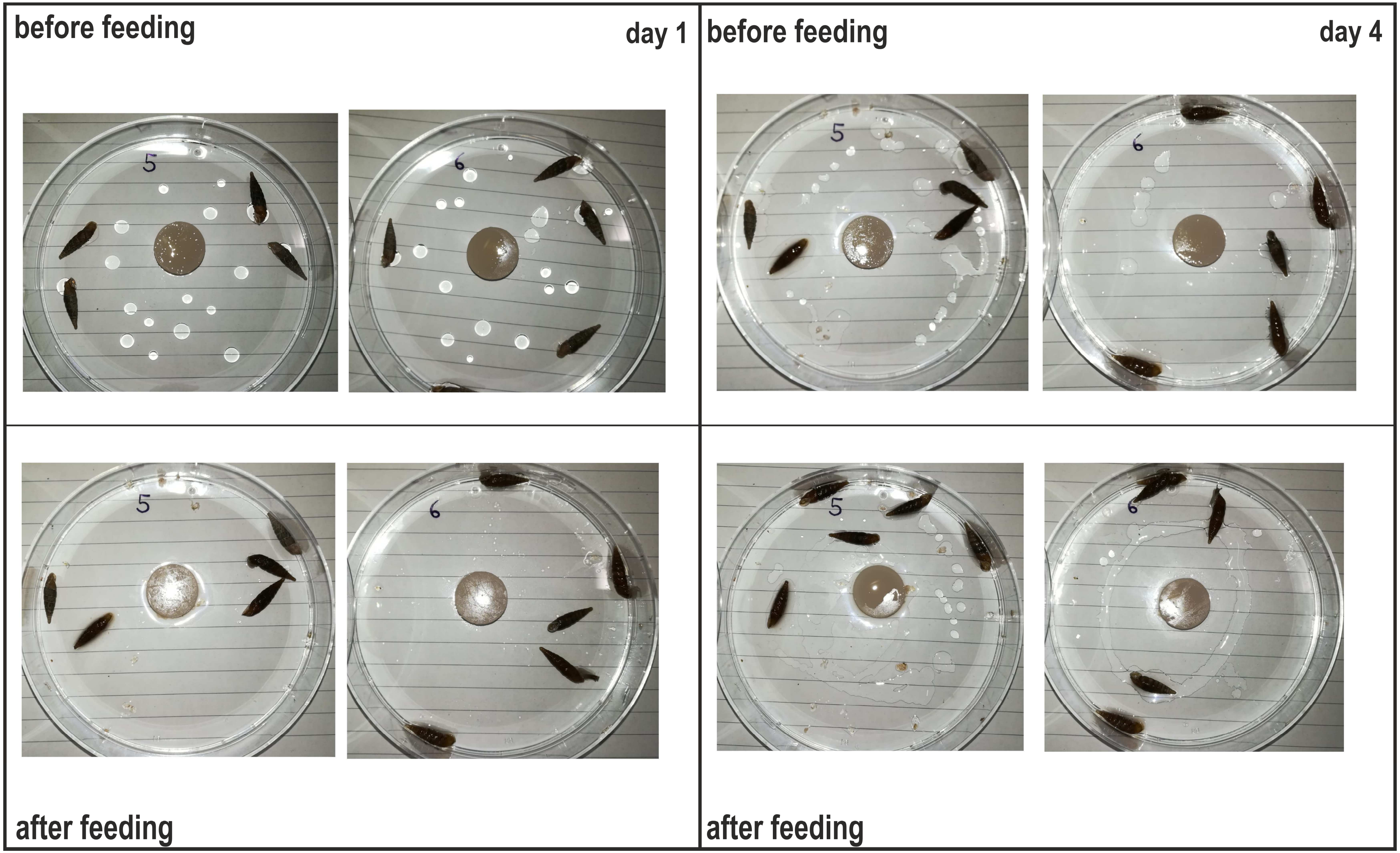
before feeding
day 1
before feeding
day 4
5
6
5
6
5
6
5
6
after feeding
after feeding

E - Cd100

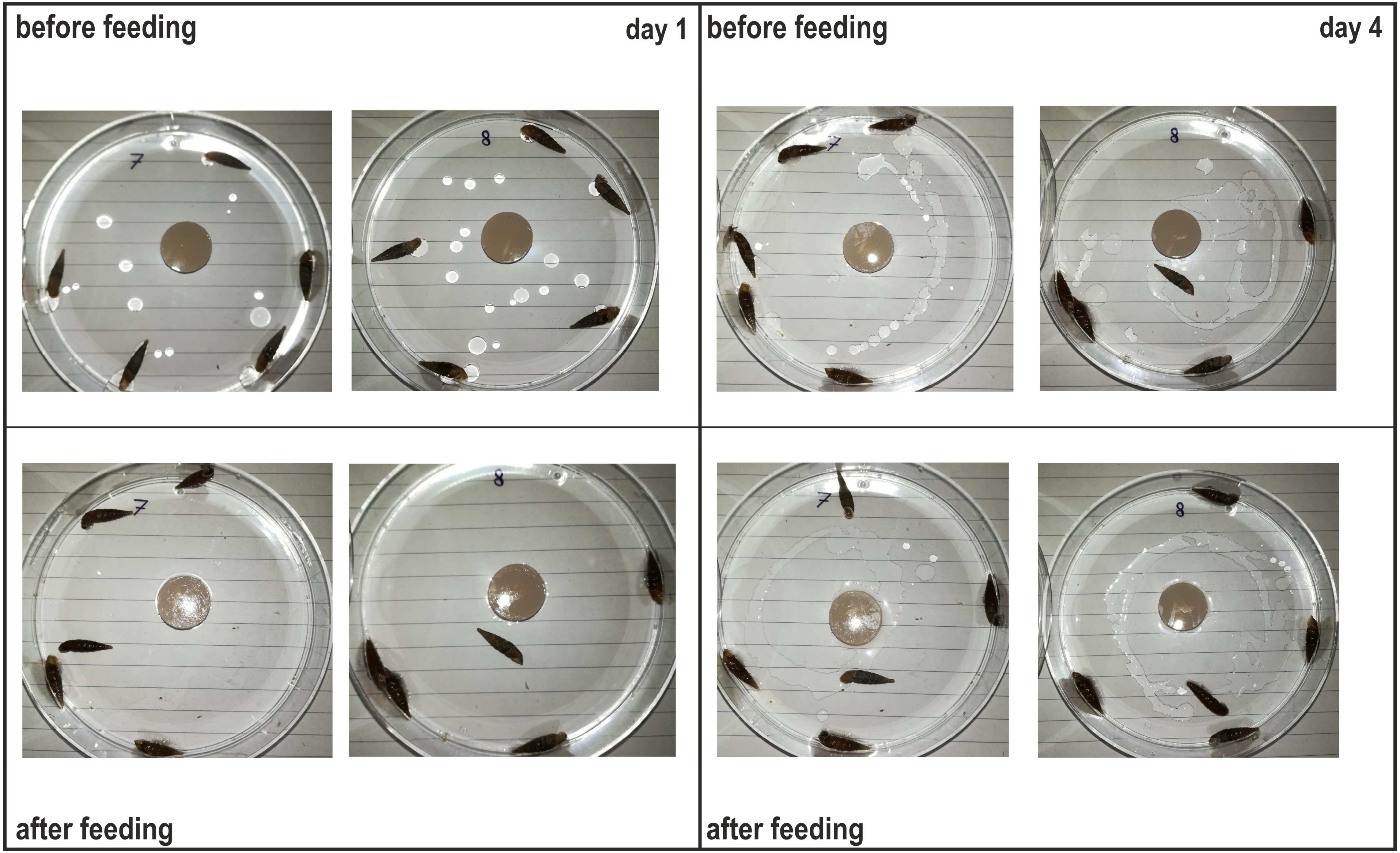
before feeding
day 1
before feeding
day 4
after feeding
after feeding

F - Cd200

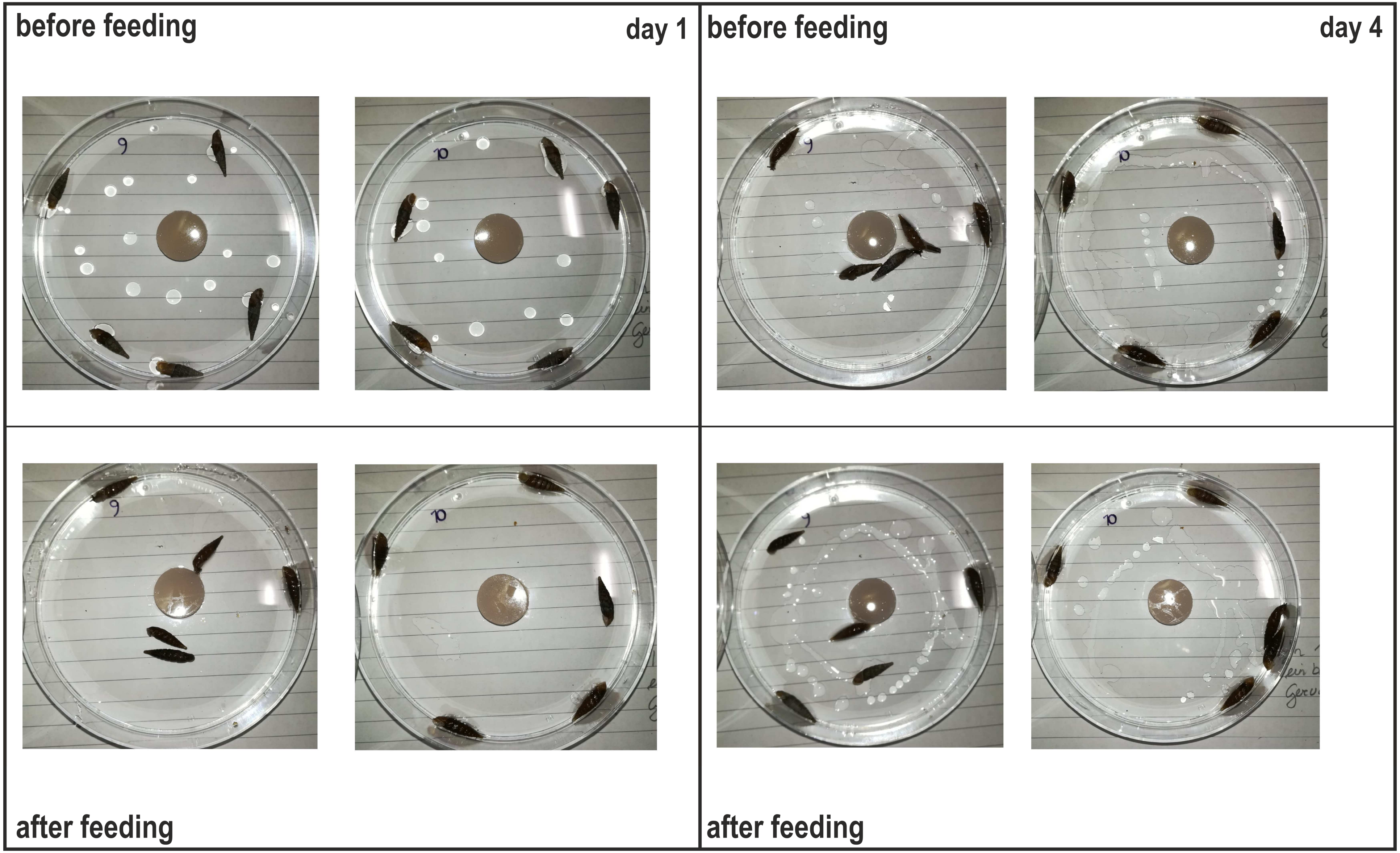
before feeding
day 1
before feeding
day 4
after feeding
after feeding
