# Supplementary material for: Cadmium Uptake, MT Gene Activation and Structure of Large-Sized Multi-Domain Metallothioneins in the Terrestrial Door Snail Alinda biplicata (Gastropoda, Clausiliidae)
Source: Int J Mol Sci. 2020 Feb 27;21(5):1631. doi: 10.3390/ijms21051631 (PMC7084494; doi:10.3390/ijms21051631)
Supplement: Supplementary file 1 [file ijms-21-01631-s001.zip › ijms-726309-supplementary PROOF/Figure S2.docx]

**Figure S2: A**lignments of (**A**) only partial md-MT peptide sequences derived from transcriptomic data (SRA: SRX 7671047) identified with a tBlastN and (**B**) PCR confirmation of the respective contigs for the 10md-MT cDNA sequence.

**A) Alignment of received contigs by Blast against the transcriptomic data set:**

alinda_biplicata_A1_DN83311_c2_g2_i1 ------GCTCASCKNTKACTGDCKSDPCKCGDNCQCGDGCTCASCKTCKCT-----NEGC 49

alinda_biplicata_B1_DN81806_c1_g1_i1 ------------CKSTKACTGDCKSDPCKCGDNCQCGVGCTCASCKNTKACTGDCKSDPC 48

alinda_biplicata_B1_DN81806_c1_g2_i1 NCQCGEGCTCASCKSTKACTGDCKSDPCKCGDNCQCGEGCTCASCKSTKACTGDCKSDPC 60

alinda_biplicata_DN108846_c0_g4_i1 -------------MSGKACTGDCKSDPCKCGANCQCGEGCTCASCKSTKACTGDCKSDPC 47

alinda_biplicata_B1_DN81806_c1_g2_i2 NCQCGEGCTCASCKSTKACTGDCKSDPCKCGDNCQCGEGCTCASCKSTKACTGDCKSDPC 60

. *************** ***** ********. *. .: *

alinda_biplicata_A1_DN83311_c2_g2_i1 KCGQECTGPATCKCAS--------------------GCSCK*- 70

alinda_biplicata_B1_DN81806_c1_g1_i1 KCGANCQCGEGCTCASCKSTKACTGDCKSDPCKCGDNCQCGEG 91

alinda_biplicata_B1_DN81806_c1_g2_i1 KCGDNCQCGVGCTCASCKSTKACTGDCKSDPCKCG-------- 95

alinda_biplicata_DN108846_c0_g4_i1 KCGDNCQCGEGCTCA---------------------------- 62

alinda_biplicata_B1_DN81806_c1_g2_i2 KCGDNCQCGEGCTCASCKSTKACTGD----------------- 86

*** :* *.**

**B) Alignment with the 10md-MT confirmed via LD PCR**

alinda_biplicata_B1_DN81806_c1_g2_i2 -------------------NCQCGEGCTCASCKSTKACTGDCKSDPCKCGDNCQCGEGCT 41

alinda_biplicata_B1_DN81806_c1_g2_i1 -------------------NCQCGEGCTCASCKSTKACTGDCKSDPCKCGDNCQCGEGCT 41

alinda_biplicata_B1_DN81806_c1_g1_i1 ------------------------------------------------------------ 0

alinda_biplicata_A1_DN83311_c2_g2_i1 ------------------------------------------------------------ 0

alinda_biplicata_DN108846_c0_g4_i1 MSGKACTGDCKSDPCKCGANCQCGEGCTCASCKSTKACTGDCKSDPCKCGDNCQCGEGCT 60

**10md_MT MSGKACTGDCKSDPCKCGANCQCGEGCTCASCKSTKACTGDCKSDPCKCGDNCQCGEGCT 60**

alinda_biplicata_B1_DN81806_c1_g2_i2 CASCKSTKACTGDCKSDPCKCGDNCQCGEGCTCASCKSTKACTGD--------------- 86

alinda_biplicata_B1_DN81806_c1_g2_i1 CASCKSTKACTGDCKSDPCKCGDNCQCGVGCTCASCKSTKACTGDCKSDPCKCG------ 95

alinda_biplicata_B1_DN81806_c1_g1_i1 ------------------------------------------------------------ 0

alinda_biplicata_A1_DN83311_c2_g2_i1 ------------------------------------------------------------ 0

alinda_biplicata_DN108846_c0_g4_i1 CA---------------------------------------------------------- 62

**10md-MT CASCKSTKACTGDCKSDPCKCGDNCQCGVGCTCASCKSTKACTGDCKSDPCKCGDNCQCG 120**

alinda_biplicata_B1_DN81806_c1_g2_i2 ------------------------------------------------------------ 86

alinda_biplicata_B1_DN81806_c1_g2_i1 ------------------------------------------------------------ 95

alinda_biplicata_B1_DN81806_c1_g1_i1 ---------------------------------------CKSTKACTGDCKSDPCKCGDN 21

alinda_biplicata_A1_DN83311_c2_g2_i1 ------------------------------------------------------------ 0

alinda_biplicata_DN108846_c0_g4_i1 ------------------------------------------------------------ 62

**10md-MT EGCTCASCKNTKACTGDCKSDPCKCGDNCQCGEGCTCASCKSTKACTGDCKSDPCKCGDN 180**

alinda_biplicata_B1_DN81806_c1_g2_i2 ------------------------------------------------------------ 86

alinda_biplicata_B1_DN81806_c1_g2_i1 ------------------------------------------------------------ 95

alinda_biplicata_B1_DN81806_c1_g1_i1 CQCGVGCTCASCKNTKACTGDCKSDPCKCGANCQCGEGCTCASCKSTKACTGDCKSDPCK 81

alinda_biplicata_A1_DN83311_c2_g2_i1 ------------------------------------------------------------ 0

alinda_biplicata_DN108846_c0_g4_i1 ------------------------------------------------------------ 62

**10md-MT CQCGVGCTCASCKNTKACTGDCKSDPCKCGANCQCGEGCTCASCKSTKACTGDCKSDPCK 240**

alinda_biplicata_B1_DN81806_c1_g2_i2 ------------------------------------------------------------ 86

alinda_biplicata_B1_DN81806_c1_g2_i1 ------------------------------------------------------------ 95

alinda_biplicata_B1_DN81806_c1_g1_i1 CGDNCQCGEG-------------------------------------------------- 91

alinda_biplicata_A1_DN83311_c2_g2_i1 ---------GCTCASCKNTKACTGDCKSDPCKCGDNCQCGDGCTCASCKTCKCTNEGCKC 51

alinda_biplicata_DN108846_c0_g4_i1 ------------------------------------------------------------ 62

**10md-MT CGDNCQCGEGCTCASCKNTKACTGDCKSDPCKCGDNCQCGDGCTCASCKTCKCTNEGCKC 300**

alinda_biplicata_B1_DN81806_c1_g2_i2 -------------------- 86

alinda_biplicata_B1_DN81806_c1_g2_i1 -------------------- 95

alinda_biplicata_B1_DN81806_c1_g1_i1 -------------------- 91

alinda_biplicata_A1_DN83311_c2_g2_i1 GQECTGPATCKCASGCSCK* 70

alinda_biplicata_DN108846_c0_g4_i1 -------------------- 62

**10md-MT GQECTGPATCKCASGCSCK* 319**
