# Supplementary material for: Cadmium Uptake, MT Gene Activation and Structure of Large-Sized Multi-Domain Metallothioneins in the Terrestrial Door Snail Alinda biplicata (Gastropoda, Clausiliidae)
Source: Int J Mol Sci. 2020 Feb 27;21(5):1631. doi: 10.3390/ijms21051631 (PMC7084494; doi:10.3390/ijms21051631)
Supplement: Supplementary file 1 [file ijms-21-01631-s001.zip › ijms-726309-supplementary PROOF/Figure S3.docx]

**Figure S3:**

**A) Alignment of all N-domains of the 9md and 10md-MT of *Alinda biplicata***, with a resulting consensus sequence for the N-terminal domain. The 9md-MT lacks the N6-domain (see Figure 4). The degree of conservation for each amino acid position is shown by red-coloured bar graphs underneath the alignment, followed below by the respective sequence logo.

Linker


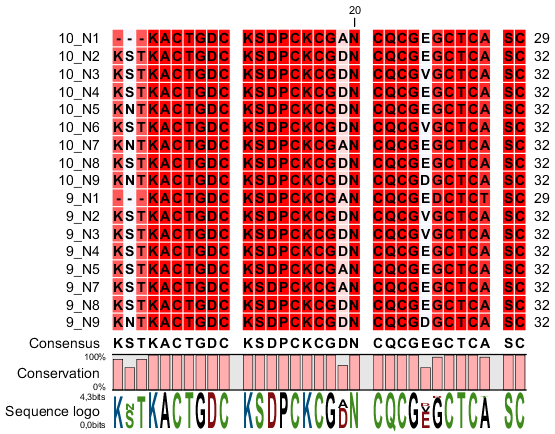


**B) Sequence coverage of the 9md-MT and 10md-MT consensus sequences of *Alinda biplicata* (Ab) with Cd- and Cu-specific MTs of helicid snails.** In the upper part a table presents % identities and e-values derived from a BlastP comparing the MTs mentioned before. For the sake of a better comparison between the md-MTs from *Alinda biplicata* and the typical two-domain MTs from *Helix pomatia* (Hp) and *Cornu aspersum* (Ca), artificial sequences for both md-MTs were generated by replacing the multiple N-domains with the consensus sequence as shown above in Figure S3A (see sequence list underneath).

| ***query*** |  | ***% identity*** | ***e-value*** |
| --- | --- | --- | --- |
| 10md-MT_ab | CdMT_Ca | 62.687 | 1.20E-23 |
| 10md-MT_ab | CdMT_Hp | 69.091 | 7.04E-22 |
| 10md-MT_ab | CuMT_Hp | 54.545 | 7.22E-20 |
| 10md-MT_ab | CuMT_Ca | 48.485 | 9.52E-17 |
| 9md-MT_Ab | CdMT_Ca | 61.194 | 4.20E-23 |
| 9md-MT_Ab | CdMT_Hp | 67.273 | 2.65E-21 |
| 9md-MT_Ab | CuMT_Hp | 54.545 | 4.46E-20 |
| 9md-MT_Ab | CuMT_Ca | 48.485 | 6.71E-17 |

>9md-MT_Ab

MSGKSTKACTGDCKSDPCKCGDNCQCGEGCTCASCKTCKCTNEACKCGQECTGPATCKCASGCSCK

>10md-MT_Ab

MSGKSTKACTGDCKSDPCKCGDNCQCGEGCTCASCKTCKCTNEGCKCGQECTGPATCKCASGCSCK

>CdMT_Hp [Genbank AF399740.1]

MSGKGKGEKCTSACRSEPCQCGSKCQCGEGCTCAACKTCNCTSDGCKCGKECTGPDSCKCGSSCSCK

>CdMT_Ca [GenBank EF152281.1]

MSGKGKGEKCTAACRNEPCQCGSKCQCGEGCTCAACKTCNCTSDGCKCGKACTGPDSCTCGSSCGCK

>CuMT_Hp [GenBank AF399741.1]

MSGRGKNCGGACNSNPCSCGNDCKCGAGCNCDRCSSCHCSNDDCKCGSQCTGSGSCKCGSACGCK

>CuMT_Ca [GenBank EF178297.2]

MSGRGQNCGGACNSNPCNCGNDCNCGTGCNCDQCSARHCSNDDCKCGSQCTRSGSCKCGNACGCK
