# Supplementary material for: Cadmium Uptake, MT Gene Activation and Structure of Large-Sized Multi-Domain Metallothioneins in the Terrestrial Door Snail Alinda biplicata (Gastropoda, Clausiliidae)
Source: Int J Mol Sci. 2020 Feb 27;21(5):1631. doi: 10.3390/ijms21051631 (PMC7084494; doi:10.3390/ijms21051631)
Supplement: Supplementary file 1 [file ijms-21-01631-s001.zip › ijms-726309-supplementary PROOF/Table S1.docx]

**Table S1:** Sequence comparison off all single domains of the 9md-MT (9N1-9N9; 9C) and 10md-MT (10N1 – 10N9, 10C) of *Alinda biplicata* by BlastN (NCBI, high similarity, default parameters). The N6 domain is missing in the 9md-MT isoform. In some cases no comparison and hence no alignment (n.a.) was found when blasting the sequences against each other.

| **domain** | **10N1** | **10N2** | **10N3** | **10N4** | **10N5** | **10N6** | **10N7** | **10N8** | **10N9** | **10C** | **9N1** | **9N2** | **9N3** | **9N4** | **9N5** | **9N7** | **9N8** | **9N9** | **9C** |
| --- | --- | --- | --- | --- | --- | --- | --- | --- | --- | --- | --- | --- | --- | --- | --- | --- | --- | --- | --- |
| **10N1** |  | 96.55 (2e-^39^) | n.a. | 95.40 (1e-^37^) | 97.70 (6e-^41^) | n.a. | 96.55 (3e-^39^) | 96.55 (3e-^39^) | n.a. | n.a. | 97.70  (5e-^41^) | 96.55 (3e-^39^) | n.a. | 96.55 (3e-^39^) | 98.85 (1e-^41^) | 96.55 (3e-^39^) | 96.55 (3e-^39^) | n.a. | n.a. |
| **10N2** | n.a. |  | 96.88 (3e-^44^) | 96.88 (3e-^44^) | 97.92 (6e-^46^) | 95.83 (1e-^42^) | 94.79 (6e-^41^) | 97.92 (6e-^46^) | 95.60 (8e-^40^) | n.a. | 94.25 (5e-^39^) | 97.92 (6e-^49^) | 95.83 (1e-^42^) | 100 (3e-^49^) | 97.92 (6e-^46^) | 94.79 (6e-^41^) | 97.92 (6e-^46^) | 95.60 (8e-^40^) | n.a. |
| **10N3** | n.a. | 96.88 (3e-^44^) |  | 97.92 (6e-^46^) | 96.88 (3e-^44^) | 98.96 (1e-^47^) | 93.75 (3e-^39^) | 96.88 (3e-^44^) | 96.70 (2e-^41^) | n.a. | n.a. | 98.96 (1e-^47^) | 96.88 (3e-^44^) | 96.88 (3e-^44^) | 96.88 (3e-^44^) | 93.75 (3e-^39^) | 96.88 (3e-^44^) | 96.70 (2e-^41^) | n.a. |
| **10N4** | 95.40 (1e-^37^) | 96.88 (3e-^44^) | 97.92 (6e-^46^) |  | 96.88 (3e-^44^) | 98.96 (1e-^47^) | 93.75 (3e-^39^) | 96.88 (3e-^44^) | 96.70 (2e-^41^) | n.a. | n.a. | 96.88 (3e-^44^) | 94.79 (6e-^41^) | 96.88 (3e-^44^) | 96.88 (3e-^44^) | 93.75 (3e-^39^) | 96.88 (3e-^44^) | 96.70 (2e-^41^) | n.a. |
| **10N5** | 97.70 (5e-^41^) | 97.92 (6e-^49^) | 96.88 (3e-^44^) | 96.88 (3e-^44^) |  | 95.83 (1e-^42^) | 96.88 (3e-^44^) | 97.92 (6e-^46^) | 95.83 (1e-^42^) | n.a. | 95.40 (1e-^37^) | 97.92 (6e-^46^) | 95.83 (1e-^42^) | 97.92 (6e-^46^) | 97.92 (6e-^46^) | 94.79 (6e-^41^) | 97.92 (6e-^46^) | 95.83 (1e-^42^) | n.a. |
| **10N6** | n.a. | 95.83 (1e-^42^) | 98.96 (1e-^47^) | 98.96 (1e-^47^) | 95.83 (1e-^42^) |  | 92.71 (1e-^37^) | 95.83 (1e-^42^) | 95.60 (8e-^40^) | n.a. | n.a. | 97.92 (6e-^46^) | 95.83 (1e-^42^) | 95.83 (1e-^42^) | 95.83 (1e-^42^) | 92.71 (1e-^37^) | 95.83 (1e-^42^) | 95.60 (8e-^40^) | n.a. |
| **10N7** | 96.55 (2e-^39^) | 94.79 (1e-^42^) | 93.75 (3e-^39^) | 93.75 (3e-^39^) | 96.88 (3e-^44^) | 92.71 (1e-^37^) |  | 96.88 (3e-^44^) | 94.79 (6e-^41^) | n.a. | 94.25 (5e-^36^) | 94.79 (6e-^41^) | 94.79 (6e-^41^) | 94.79 (6e-^41^) | 96.88 (3e-^44^) | 97.92 (6e-^46^) | 96.88 (3e-^44^) | 94.79 (6e-^41^) | n.a. |
| **10N8** | 96.55 (2e-^39^) | 97.92 (6e-^41^) | 96.88 (3e-^44^) | 96.88 (3e-^44^) | 97.92 (6e-^46^) | 95.83 (1e-^42^) | 96.88 (3e-^44^) |  | 97.80 (4e-^43^) | n.a. | 94.25 (5e-^36^) | 97.92 (6e-^46^) | 95.83 (1e-^42^) | 97.92 (6e-^46^) | 97.92 (6e-^46^) | 96.88 (3e-^44^) | 100 (3e-^49^) | 97.80 (4e-^43^) | n.a. |
| **10N9** | n.a. | 97.92 (6e-^49^) | 96.70 (2e-^41^) | 96.70 (2e-^41^) | 95.83 (1e-^42^) | 95.60 (8e-^40^) | 94.79 (6e-^41^) | 97.80 (4e-^43^) |  | n.a. | n.a. | 95.60 (8e-^40^) | 93.41 (2e-^36^) | 95.60 (8e-^40^) | 95.60 (8e-^40^) | 94.51 (4e-^38^) | 97.80 (4e-^43^) | 100 (3e-^49^) | n.a. |
| **10C** | n.a. | n.a. | n.a. | n.a. | n.a. | n.a. | n.a. | n.a. | n.a. |  | n.a. | n.a. | n.a. | n.a. | n.a. | n.a. | n.a. | n.a. | 98.92 (6e-^42^) |
| **9N1** | 97.70 (5e-^41^) | 94.25 (6e-^39^) | n.a. | n.a. | 95.40 (1e-^37^) | n.a. | 94.25 (6e-^36^) | 94.25  (6e-^36^) | n.a. | n.a. |  | 94.25 (6e-^36^) | n.a. | 94.25 (6e-^36^) | 96.55 (3e-^39^) | 94.25 (6e-^36^) | 94.25 (6e-^36^) | n.a. | n.a. |
| **9N2** | 96.55 (2e-^39^) | 97.92 (6e-^49^) | 98.96 (1e-^47^) | 96.88 (3e-^44^) | 97.92 (6e-^46^) | 97.92 (6e-^46^) | 94.79 (6e-^41^) | 97.92 (6e-^46^) | 95.60 (8e-^40^) | n.a. | 94.25 (5e-^36^) |  | 97.92 (6e-^46^) | 97.92 (6e-^46^) | 97.92 (6e-^46^) | 94.79 (6e-^41^) | 97.92 (6e-^46^) | 95.60 (8e-^40^) | n.a. |
| **9N3** | n.a. | 95.83 (1e-^42^) | 96.88 (3e-^44^) | 94.79 (6e-^41^) | 95.83 (1e-^42^) | 95.83 (1e-^42^) | 94.79 (6e-^41^) | 95.83 (1e-^42^) | 93.41 (2e-^36^) | n.a. | n.a. | 97.92 (6e-^46^) |  | 95.83 (1e-^42^) | 95.83 (1e-^42^) | 94.79 (6e-^41^) | 95.83 (1e-^42^) | 93.41 (2e-^36^) | n.a. |
| **9N4** | 96.55 (2e-^39^) | 100 (3e-^49^) | 96.88 (3e-^44^) | 96.88 (3e-^44^) | 97.92 (6e-^46^) | 95.83 (1e-^42^) | 94.79 (6e-^41^) | 97.92 (6e-^46^) | 95.60 (8e-^40^) | n.a. | 94.25 (5e-^36^) | 97.92 (6e-^46^) | 95.83 (1e-^42^) |  | 97.92 (6e-^46^) | 94.79 (6e-^41^) | 97.92 (6e-^46^) | 95.60 (8e-^40^) | n.a. |
| **9N5** | 98.85 (1e-^41^) | 97.92 (6e-^49^) | 96.88 (3e-^44^) | 96.88 (3e-^44^) | 97.92 (6e-^46^) | 95.83 (1e-^42^) | 96.88 (3e-^44^) | 97.92 (6e-^46^) | 95.60 (8e-^40^) | n.a. | 96.55 (2e-^39^) | 97.92 (6e-^46^) | 95.83 (1e-^42^) | 97.92 (6e-^46^) |  | 96.88 (3e-^44^) | 97.92 (6e-^46^) | 95.60 (8e-^40^) | n.a. |
| **9N7** | 96.55 (2e-^39^) | 94.79 (1e-^42^) | 93.75 (3e-^39^) | 93.75 (3e-^39^) | 94.79 (6e-^41^) | 92.71 (1e-^37^) | 97.92 (6e-^46^) | 96.88 (3e-^44^) | 94.51 (4e-^38^) | n.a. | 94.25 (5e-^36^) | 94.79 (6e-^41^) | 94.79 (6e-^41^) | 94.79 (6e-^41^) | 96.88 (3e-^44^) |  | 96.88 (3e-^44^) | 94.51  (4e-^38^) | n.a. |
| **9N8** | 96.55 (2e-^39^) | 97.92 (6e-^49^) | 96.88 (3e-^44^) | 96.88 (3e-^44^) | 97.92 (6e-^46^) | 95.83 (1e-^42^) | 96.88 (3e-^44^) | 100 (3e-^49^) | 97.80 (4e-^43^) | n.a. | 94.25 (5e-^36^) | 97.92 (6e-^46^) | 95.83 (1e-^42^) | 97.92 (6e-^46^) | 97.92 (6e-^46^) | 94.51 (4e-^38^) |  | 97.80 (4e-^43^) | n.a. |
| **9N9** | n.a. | 95.60  (8e-^40^) | 96.70 (2e-^41^) | 96.70 (2e-^41^) | 95.83 (1e-^42^) | 95.60 (8e-^40^) | 94.79 (6e-^41^) | 97.80 (4e-^43^) | 100 (3e-^49^) | n.a. | n.a. | 95.60 (8e-^40^) | 93.41 (2e-^36^) | 95.60 (8e-^40^) | 95.60 (8e-^40^) | 96.88 (3e-^44^) | 97.80 (4e-^43^) |  | n.a. |
| **9C** | n.a. | n.a. | n.a. | n.a. | n.a. | n.a. | n.a. | n.a. | n.a. | 98.92 (6e-^42^) | n.a. | n.a. | n.a. | n.a. | n.a. | n.a. | n.a. | n.a. |  |
