# Supplementary material for: Cadmium Uptake, MT Gene Activation and Structure of Large-Sized Multi-Domain Metallothioneins in the Terrestrial Door Snail Alinda biplicata (Gastropoda, Clausiliidae)
Source: Int J Mol Sci. 2020 Feb 27;21(5):1631. doi: 10.3390/ijms21051631 (PMC7084494; doi:10.3390/ijms21051631)
Supplement: Supplementary file 1 [file ijms-21-01631-s001.zip › ijms-726309-supplementary PROOF/Table S2.docx]

**Table S2:** Characterization of gene specific primers **(A)** and PCR parameters **(B)** used for amplification of the 9md and 10md-MT mRNA (n=5 individuals) and genomic DNA sequences (n=3; samples derived from pooled individuals) in *Alinda biplicata*.

**A)** List of gene specific primers

| ***Name*** | ***Sequence 5’-3’*** | ***Tm [°C]*** |
| --- | --- | --- |
| MT3_S | TCC TCG TAC CGT ATC TCA GC | 60.5 |
| MT3_AS | ACG CTC AAT GTC GTC TTA TGT TGC | 62 |

**B)** PCR cycling conditions for 9md and 10md-MT mRNA and *MT* gene amplification

| ***mRNA sequence – Advantage Polymerase*** | | | | ***gDNA sequence – SuperFi Green PCR MM*** | | | |
| --- | --- | --- | --- | --- | --- | --- | --- |
| ***Step*** | ***Temp. [°C]*** | ***time*** | ***cycle*** | ***Step*** | ***Temp. [°C]*** | ***time*** | ***cycle*** |
| Denaturation | 95 | 1 min | 1x | Denaturation | 98 | 5 min | 1x |
| Denaturation | 95 | 30 sec | 30x | Denaturation | 98 | 10 sec | 35x |
| Annealing | 58 | 30 sec |  | Annealing | 58 | 10 sec |  |
| Extension | 68 | 1 min |  | Extension | 72 | 5 min 30sec |  |
| Final Extension | 68 | 5 min | 1x | Final Extension | 72 | 5 min | 1x |
